# Quantifying and Leveraging Interfacial Amine Reactivity in Block Copolymer Nanoparticles for Advanced Material Design

**DOI:** 10.1002/smsc.70295

**Published:** 2026-05-15

**Authors:** Aharon Steffè, Beatrice Rosetti, Stefano Valente, Maria Sbacchi, Federica Battistin, Paolo Tecilla, Pierangelo Gobbo

**Affiliations:** ^1^ Department of Chemical and Pharmaceutical Sciences University of Trieste Trieste Italy; ^2^ National Interuniversity Consortium of Materials Science and Technology, Unit of Trieste Firenze Italy

## Abstract

The rational design of functional nanomaterials requires precise control over molecular architecture and interfacial reactivity. Here, we report the synthesis of amine‐functionalized block copolymer nanoparticles via aqueous polymerization‐induced self‐assembly, combining synthetic efficiency with rigorous characterization to bridge molecular design and macroscopic functionality. The incorporation of 2‐aminoethyl methacrylate into a macrochain transfer agent enabled the formation of stable, monodisperse nanoparticles while providing quantifiable interfacial amines for postassembly modification. Near‐quantitative conjugation with fluorescein isothiocyanate (86% yield) or thermoresponsive poly(*N*‐isopropylacrylamide) (91% efficiency) demonstrated the platform versatility, with the latter exhibiting a tunable lower critical solution temperature transition at 41°C. Furthermore, the nanoparticles could be self‐assembled into crosslinked colloidosomes, highlighting their potential as modular building blocks for the fabrication of hierarchical architectures. This work establishes a paradigm for engineering functional polymeric nanomaterials with tailored properties, offering transformative opportunities in drug delivery, diagnostics, and nanoreactor design. By linking molecular‐scale precision to predictable performance, our approach advances the field from empirical optimization to true molecular engineering of polymeric nanoparticles.

## Graphical abstract

Amine‐functionalized block copolymer nanoparticles were synthesized via RAFT‐PISA with rigorous quantification of interfacial amine accessibility. The surface‐reactive amines served as modular handles for postassembly conjugation with fluorescent dyes and thermoresponsive polymers, conferring optical trackability and stimuli‐responsive behaviors. Further hierarchical assembly into crosslinked, water‐stable colloidosomes demonstrates the nanoparticles as precision‐engineered building blocks for advanced functional architectures.

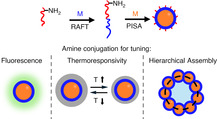

## 1 Introduction

The quest to create functional nanomaterials with precisely controlled architectures, functionalities, and reactivity has become one of the most vibrant ventures in polymer science and colloidal engineering. At the heart of this endeavor lies a fundamental challenge: how to bridge the gap between molecular‐scale design and macroscopic functionality.

Polymerization‐induced self‐assembly (PISA) has emerged as a uniquely powerful solution to this challenge, combining the synthetic precision of controlled living radical polymerization with a spontaneous block copolymer self‐assembly [1, 2, 3, 4]. By growing insoluble blocks from soluble macromolecular chain transfer agents (macroCTAs) directly in solution, PISA enables the one‐pot synthesis of well‐defined nanoparticles at high concentrations, which represents a transformative advance over traditional postpolymerization processing methods. Through careful optimization of parameters including block ratios, solvent composition, and polymerization temperature, researchers can now access an extraordinary diversity of morphologies spanning spheres, worms, vesicles, and even more complex architectures like framboidal or multicompartmental structures [5, 6, 7, 8, 9]. What truly distinguishes PISA from conventional nanoparticle fabrication methods are three key advantages: (1) a streamlined synthesis with fewer steps, (2) the production of nano‐object dispersions at unprecedented concentrations (e.g., up to 50 wt% versus 0.5–1 wt% for nanoprecipitation), and (3) the inherent capacity for in situ functional group incorporation, enabling scalable production of tailored nanomaterials [10]. These features position PISA not merely as a shape‐control technique, but as a versatile platform for engineering smart nanomaterials with programable surface chemistry and functionality.

To date, significant progress has been made in expanding PISA’s scope, including the development of different types of polymerization stimuli (e.g., light, redox chemistry, enzyme catalysis, microwaves etc*.*), the exploration of novel monomer systems for morphology control, and the design of chemically crosslinked nano‐objects to withstand purification protocols and further use [11]. Furthermore, PISA has been leveraged for polymerizing sensitive monomers and encapsulating molecular cargos, paving the way for applications in drug delivery [12]. However, critical challenges remain, particularly in creating reactive nanoparticle coronas or cores and in characterizing their postassembly reactivity, an area that remains essentially unexplored despite its potential to unlock advanced functionalities. In particular, the ability to install reactive handles directly onto nanoparticle coronas during their formation would open exciting possibilities for postassembly modification, stimuli‐responsive behavior, and hierarchical organization. Such molecular‐level control is especially compelling for applications requiring precise interfacial engineering, including targeted drug delivery systems where ligand density governs cellular uptake [13, 14], nanoreactors where catalytic site accessibility determines efficiency [15, 16, 17, 18, 19], or protocell constructs where surface chemistry mediates compartmental communication [20, 21, 22, 23, 24].

Among the various functional groups that could be incorporated via PISA, primary amines hold special significance due to their unparalleled versatility in bioconjugation chemistry and materials modification. For instance, the protonated form of 2‐aminoethyl methacrylate (AEMA) permits reversible addition‐fragmentation chain‐transfer (RAFT) polymerization [25, 26, 27], and its cationic character confers additional advantages for biomedical applications, including enhanced membrane interaction and endosomal escape properties [28, 29]. The deprotonated amine, on the other hand, enables diverse coupling reactions including isothiocyanate conjugation, NHS‐activated ester amidation, Michael‐type additions, epoxy‐amine addition, and much more. This dual nature of AEMA makes the corresponding amine‐functionalized nanoparticles challenging to synthesize, but ideal platforms for creating multifunctional materials through sequential modification strategies.

Our work presents the aqueous‐dispersed PISA synthesis of amine‐functionalized polymeric nanoparticles that combines synthetic efficiency with remarkable characterization rigor. At its core lies a carefully designed random copolymer stabilizer, poly(glycerol monomethacrylate‐co‐2‐aminoethyl methacrylate) (p(GMA‐AEMA)), which integrates steric stabilization and amine reactivity in a single‐component system. We coupled this design with a rigorous characterization methodology to quantify the number of accessible amines at room and high temperature. By combining this molecularly precise stabilizer with 2‐hydroxypropyl methacrylate (HPMA) and in situ crosslinking using ethylene glycol dimethacrylate (EGDMA), we achieved amine‐functionalized nanoparticles that maintained colloidal stability even after solvent removal through lyophilization, while preserving well‐defined interfacial reactivity.

While previous reports have demonstrated the incorporation of primary amines in PISA‐derived nanoparticles, the most directly relevant example employs AEMA primarily to validate polymerization compatibility and to characterize the resulting cationic colloidal behavior, without exploiting the reactive amines for postassembly modification or hierarchical structuring [26]. In contrast, the present work is designed from the outset to access surface‐reactive nanobuilding blocks that can be directly exploited for postassembly modification and hierarchical structuring. The primary amines are not introduced as a compositional variation, but as quantitatively characterized, chemically accessible handles that enable modular interfacial modification with predictable outcomes. In fact, the power of our approach becomes evident in our demonstration of two different nanoparticle functionalization pathways: (1) near‐quantitative (86% yield) fluorescein conjugation via amine‐isothiocyanate chemistry for optical tracking, and (2) grafting of thermoresponsive poly(*N*‐isopropylacrylamide) (p(NIPAM)) through NHS‐activated ester coupling (91% efficiency), yielding nanoparticles with thermal transitions (∼10% diameter change at lower critical solution temperature—LCST). These results establish new benchmarks for interfacial postassembly modification in PISA systems, moving toward quantitative structure‐function relationships.

Perhaps most importantly, our reactivity control enables previously unattainable hierarchical microarchitectures stable in water. Using the precisely characterized nanoparticles as building blocks, we demonstrate the formation of colloidosomes through Pickering emulsion templating, a capability directly enabled by our fundamental understanding of interfacial amine reactivity. The surface‐accessible amines simultaneously drove covalent crosslinking and fluorescent labeling of the self‐assembled shells, yielding covalently stabilized structures that could be transferred intact into water by dialysis. Unlike previous PISA‐based Pickering emulsion systems, this crosslinking step is not merely a functionalization strategy, but the essential prerequisite that permits transfer into a fully aqueous environment, where the colloidosomes exhibited temperature‐induced contractility and selective permeability governed by the membrane molecular weight cut‐off (MWCO). The ability to produce stable, responsive, and trackable colloidosomes stable in water from precisely engineered nanoparticle building blocks represents a level of structural control that remains largely unexplored in PISA‐derived systems.

By integrating streamlined synthesis with rigorous interfacial characterization, this work provides more than just a new polymeric nanoparticle platform, it establishes a blueprint for engineering functional polymeric nanomaterials where molecular‐scale precision enables predictable performance across length scales. In doing so, we shift the focus of amine‐containing PISA nanoparticles from morphological design to chemical programmability and postassembly reactivity, opening new directions across diverse applications spanning drug delivery to diagnostic systems, nanoreactor design, microreactor technologies, and protocell engineering. More fundamentally, our approach moves the field beyond empirical optimization toward true molecular engineering of polymeric nanomaterials, where chemical design directly dictates hierarchical organization and macroscopic function.

## 2 Results and Discussion

### 2.1 PISA of Amine‐Functionalized Block Copolymer Crosslinked Nanoparticles and Their Characterization

**FIGURE 1:**
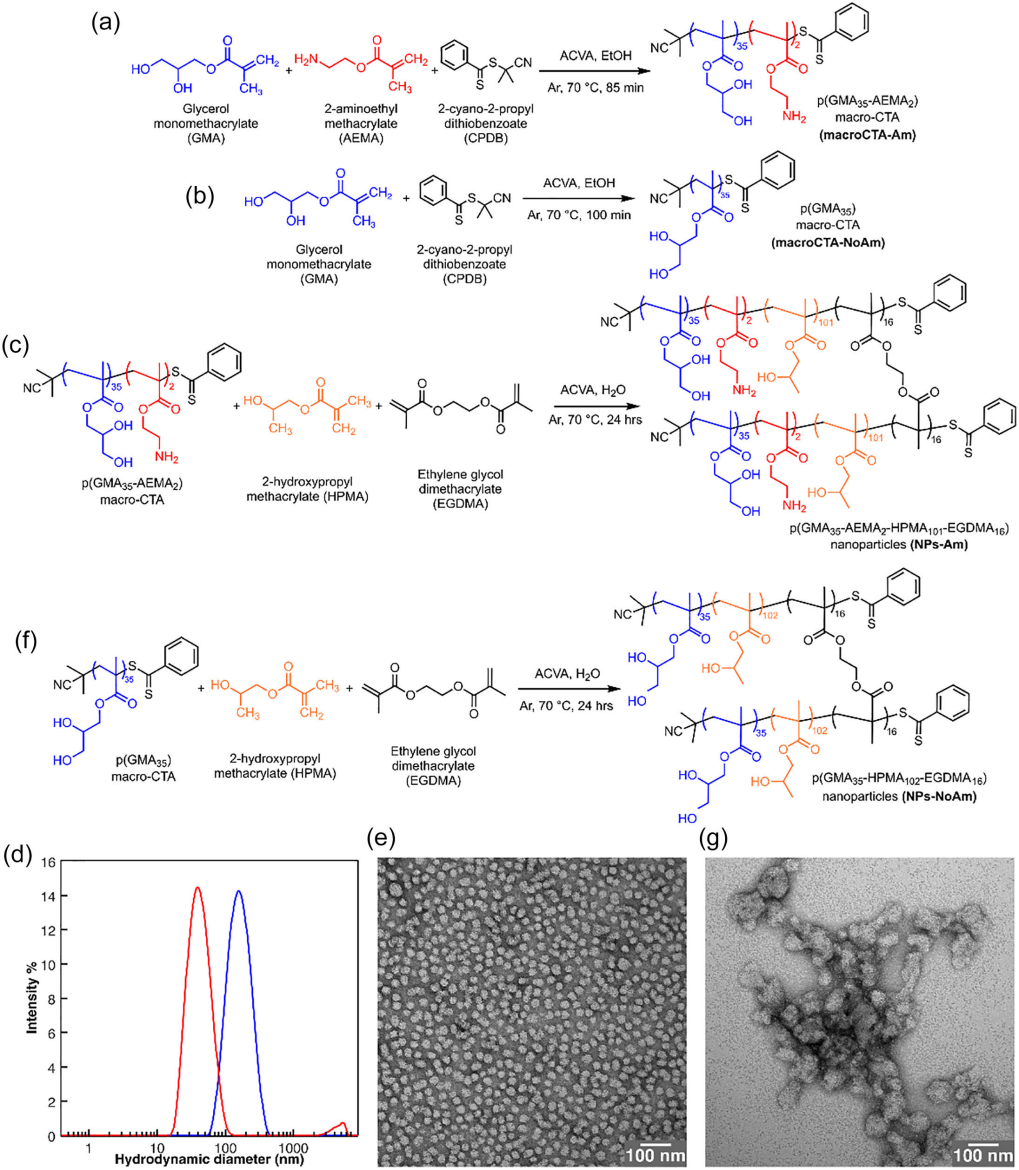
(a) Reaction scheme of the synthesis of macroCTA‐Am via RAFT polymerization. (b) Reaction scheme of the synthesis of macroCTA‐NoAm via RAFT polymerization. (c) Reaction scheme of the synthesis of NPs‐Am via RAFT‐PISA approach. (d) DLS intensity‐average hydrodynamic diameter distribution of a dispersion of NPs‐Am (0.2 mg mL^−1^ in Milli‐Q water, red plot) or of NPs‐NoAm (blue plot). (e) Representative TEM images of NPs‐Am. (f) Reaction scheme of the synthesis of NPs‐NoAm via RAFT‐PISA approach. (g) Representative TEM images of NPs‐NoAm.

In order to prepare amine‐functionalized copolymer crosslinked nanoparticles, first a p(GMA_35_‐AEMA_2_) macroCTA (macroCTA‐Am) was synthesized via RAFT polymerization using 2‐cyano‐2‐propyl dithiobenzoate (CPDB) as the RAFT agent and 4,4′‐azobis(4‐cyanopentanoic acid) (ACVA) as the thermal initiator, adapting a literature procedure (Figure 1a, Section S2.1) [6]. To preserve the end‐group of the polymer chains and prevent recombination, the RAFT polymerization reaction was quenched at approximately 50%–60% of GMA conversion. Following purification by precipitation in ice‐cold dichloromethane (CH_2_Cl_2_), ^1^H‐NMR analysis of the purified polymer indicated a monomer conversion of 55%, with a GMA degree of polymerization (DP) of 35, an AEMA DP of 2, and an estimated number‐average molecular weight (*M*_*n*_) of 6,022 g mol^−1^ (Figures S1 and S2). Unfortunately, strong interactions between the GMA‐based polymers and the stationary phase prevented accurate molecular weight determination by tetra‐detection gel permeation chromatography (TD‐GPC) in both aqueous and *N*, *N*‐dimethylformamide systems.

These interactions, likely arising from the amphiphilic character of the polymer and the high number of hydroxyl groups, hindered proper size‐exclusion separation and precluded meaningful data acquisition. The same polymer was also insoluble in tetrahydrofuran.

The Kaiser test was employed to confirm the number of amine groups *per* polymer chain and probe their accessibility for reactivity (Section S2.2) [30]. Under standard test conditions (120°C, 10 min), despite the low DP of AEMA, the primary amine content was determined to be 80 ± 9 μmol g^–1^, corresponding to less than one amine per polymer chain [31]. Since nanoparticle conjugation reactions typically occur at room temperature (vide infra), we further investigated amine accessibility under milder conditions. At 25°C for 10 min, the amount of reactive amines decreased to 43 ± 2 μmol g^–1^, and extending the reaction time to 24 h at 25°C yielded a comparable value (56 ± 10 μmol g^–1^, Table S1). These results indicate low amine accessibility under both standard and ambient conditions, which we attribute to steric hindrance and/or functional group interference. As a control experiment, we synthesized a p(GMA_35_) macroCTA (macroCTA‐NoAm) without AEMA incorporation (Figure 1b, Section S2.3) [6]. Purification of the crude polymer by precipitation in CH_2_Cl_2_ yielded a product with 56% GMA conversion, a GMA of 35, and an estimated *M*_*n*_ of 5,810 g mol^‒^^1^, as determined by ^1^H‐NMR spectroscopy (Figures S3 and S4). As observed for the previous macroCTA, the TD‐GPC analysis of macroCTA‐NoAm was prevented by insufficient solubility in common eluents or interaction with the columns. Consistent with the absence of AEMA, the Kaiser test confirmed no detectable amine groups (Table S1). Overall, these results demonstrate that, even at low amine loadings, the Kaiser test provides a sensitive probe for the presence of accessible primary amine functionalities.

The macroCTA‐Am was then used for the in situ RAFT aqueous dispersion polymerization of HPMA and EGDMA (crosslinker) to produce well‐defined, amine‐functionalized nanoparticles via PISA, with ACVA as the initiator (Figure 1c). Notably, EGDMA was added after 5 h rather than being premixed with HPMA at the start of the polymerization (Section S2.4). This approach prevented the formation of insoluble aggregates, which occurred when both monomers were introduced simultaneously at the beginning of the polymerization reaction. After polymerization, ^1^H‐NMR analysis indicated 81% of monomer conversion, with a DP of 101 for HPMA and 16 for EGDMA (Figure S5).

The resulting turbid nanoparticle dispersion was analyzed directly by dynamic light scattering (DLS) and ζ‐potential measurements across pH 4.0–12.0. When interfacial amines were protonated, nanoparticles remained monodispersed and stable in solution with an average hydrodynamic diameter of 49.3 ± 3.5 nm and ζ‐potential of +16.7 ± 1.3 mV (Figure S6a). At pH 10.0, the nanoparticles reached their isoelectric point, causing aggregation and flocculation. Above pH 11, the nanoparticles acquired a ζ‐potential of −25.3 ± 1.4 mV and a hydrodynamic diameter of 45.9 ± 2.3 nm, regaining their stability. These results agree with prior reports on similar systems [19]. Importantly, in carbonate buffer (10 mM, pH 8.5) commonly used for conjugation protocols (vide infra), the nanoparticles were stable with a hydrodynamic diameter of 66.5 ± 3.3 nm and ζ‐potential −23.5 ± 1.7 mV. These conditions are optimal for interfacial reactivity since the interfacial amines are deprotonated and available as nucleophiles, while the nanoparticles remain nonaggregated. Transmission electron microscopy (TEM) imaging further confirmed spherical, monodisperse nanoparticles with an average diameter of 28.1 ± 3.7 nm (Figures 1e and S7a).

To confirm the specific role of amine functionalities, we prepared control nanoparticles under identical PISA conditions using macroCTA‐NoAm (Figure 1f, Section S2.5). ^1^H‐NMR confirmed nearly equivalent polymerization efficiency (79% conversion, Figure S8) yielding p(GMA_35_‐HPMA_102_‐EGDMA_16_) nanoparticles (NPs‐NoAm). However, the absence of amines resulted in fundamentally different colloidal behavior. pH‐dependent measurements (pH 4.0–12.0) revealed stable nanoparticles with an average hydrodynamic diameter of 93.2  ± 6.1 nm and ζ‐potential of −23.6 ± 3.2 mV across all tested conditions, showing no pH‐dependent changes (Figure S6b). These control nanoparticles exhibited greater aggregation tendency in Milli‐Q water, with DLS and TEM imaging revealing aggregation and irregularly shaped particles with larger diameters (71.0 ±  25.3 nm) (Figures 1d,g and S7b). This contrasts with the amine‐containing nanoparticles, which exhibited pH‐responsive charge switching and maintained uniform morphology, confirming that interfacial amines provide both surface charge modulation and enhanced colloidal stability through electrostatic stabilization.

The accessibility of primary amines in the p(GMA_35_‐AEMA_2_‐HPMA_101_‐EGDMA_16_) nanoparticles (NPs‐Am) was evaluated via the Kaiser test using the NPs‐NoAm as a reference. At 120°C, the nanoparticles contained 48 ± 13 μmol g^–1^ of accessible primary amines. When tested at room temperature, the values decreased to 25 ±  1 μmol g^–1^ (10 min) and 27 ± 2 μmol g^–1^ (24 h, Table S2). As expected, due to a marked increase in polymer molecular weight, all measured values were lower than those obtained for the macroCTA‐NoAm precursor.

Taken together, these results demonstrate that surface amines are not only readily available for interfacial reactivity, but also play a crucial structural role in PISA, guiding the formation of stable, uniform, and discrete spherical nanoparticles. Their function extends beyond providing reactive moieties for further functionalization (vide infra), underscoring their dual importance in both reactivity and nanoparticle assembly.

### 2.2 Modification of Nanoparticle Physicochemical Properties via Controlled Conjugation Reactions

To demonstrate the versatility of interfacial reactive amines for the rational design of polymeric nanoparticles with tailored functionalities, we developed a conjugation strategy to achieve two distinct objectives: (1) the preparation of fluorescent nanoparticles by coupling fluorescein isothiocyanate (FITC), and (2) the introduction of thermoresponsive properties through conjugation with poly(*N*‐isopropylacrylamide‐*co*‐*N*‐acryloxy succinimide) (p(NIPAM_51_‐AANHS_3_)) polymer. This approach highlights the flexibility of reactive amines in conferring either optical or stimuli‐responsive behavior to nanoparticle systems.

**FIGURE 2:**
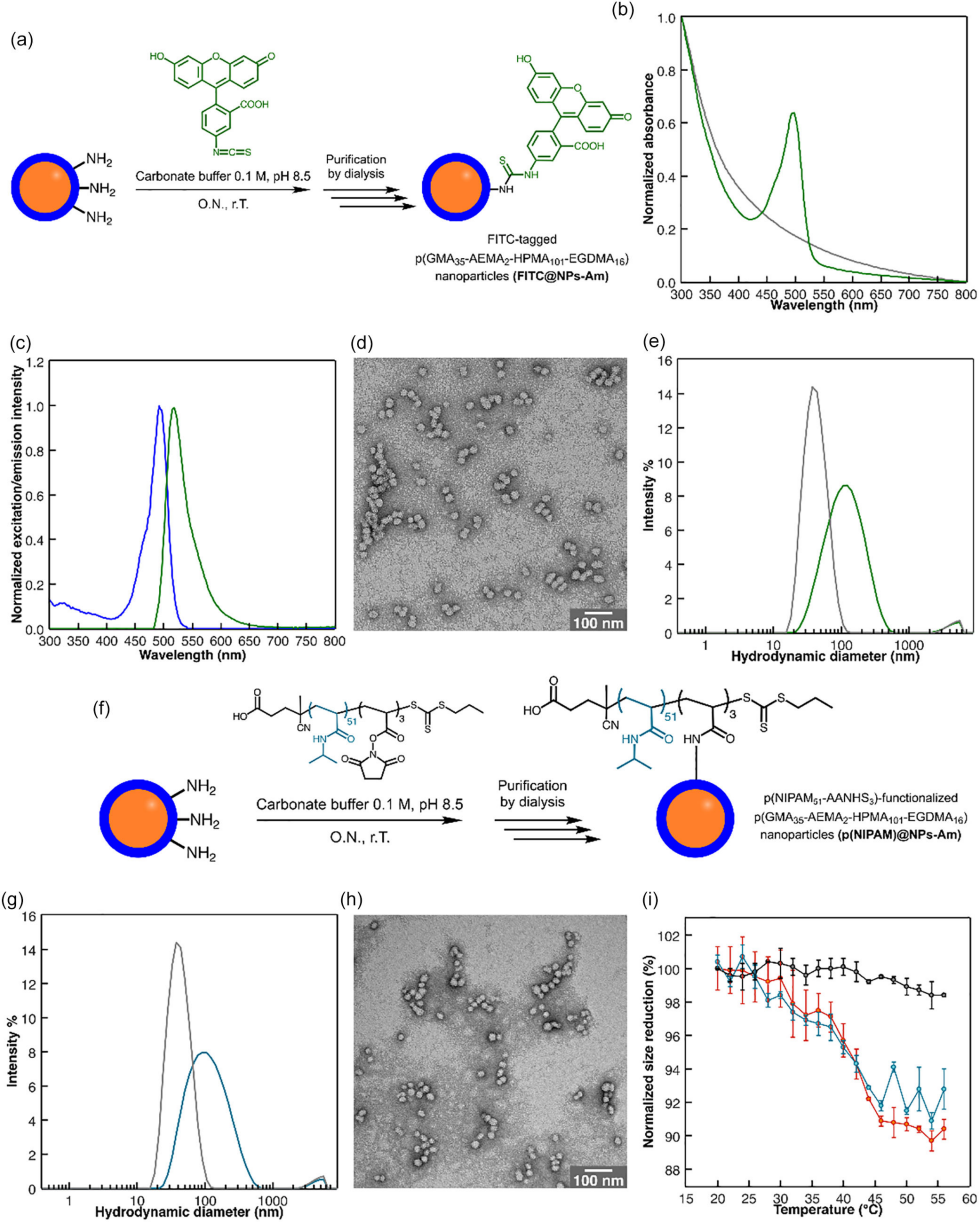
(a) Reaction scheme describing the functionalization of NPs‐Am with a fluorescent molecular probe (fluorescein isothiocyanate, FITC). (b) UV–vis spectra of a dispersion of FITC@NPs‐Am (1 mg mL^−1^ in Milli‐Q water, green plot) and of the same nanoparticles before functionalization with FITC (gray plot). (c) Excitation (blue plot) and emission (green plot) spectra of FITC@NPs‐Am (0.3 mg  mL^−1^ in Milli‐Q water). The excitation spectrum was recorded by monitoring emission at 550 nm, while the emission spectrum was obtained with excitation fixed at 480 nm. Characteristic FITC excitation and emission peaks are observed, confirming successful fluorescent labeling of the nanoparticles. (d) TEM image of FITC@NPs‐Am. (e) DLS intensity‐average hydrodynamic diameter distributions of a dispersion of pristine NPs‐Am (0.2 mg mL^−1^ in Milli‐Q water, gray plot) and of the same nanoparticles after functionalization with FITC (green plot). (f) Reaction scheme describing the functionalization of NPs‐Am with p(NIPAM_51_‐AANHS_3_). (g) DLS intensity‐average hydrodynamic diameter distributions of a dispersion of pristine NPs‐Am (0.2 mg mL^−1^ in Milli‐Q water, gray plot) and of the same nanoparticles after functionalization with p(NIPAM_51_‐AANHS_3_) polymer (light blue plot). (h) TEM image of p(NIPAM_51_‐AANHS_3_)‐functionalized p(GMA_35_‐AEMA_2_‐HPMA_101_‐EGDMA_16_) nanoparticles (p(NIPAM)@NPs‐Am). (i) Plot showing the temperature‐dependent changes in the hydrodynamic diameter of p(NIPAM)@NPs‐Am (heating cycle, red plot; cooling cycle, light blue plot) compared to pristine nanoparticles (gray plot). Error bars: standard deviation on three consecutive measurements performed on the same sample.

FITC‐tagged nanoparticles were synthesized by reacting NPs‐Am with FITC in carbonate buffer (100 mM, pH 8.5) overnight (Figure 2a, Section S2.6). Following conjugation, excess FITC was removed through sequential dialysis first against 100 mM aqueous NaCl solution for 48 h, followed by Milli‐Q water for an additional 48 h.

^1^H‐NMR analysis confirmed the absence of excess, free FITC (Figure S9), demonstrating an effective purification method, but was unable to detect the conjugated FITC due to its low concentration. Successful labeling was demonstrated by acquiring the UV–vis and fluorescence spectra of purified FITC‐tagged p(GMA_35_‐AEMA_2_‐HPMA_101_‐EGDMA_16_) nanoparticles (FITC@NPs‐Am), which showed the characteristic FITC absorbance maximum at 491 nm and corresponding emission at 517 nm (Figure 2b,c). Moreover, the Kaiser test demonstrated significant amine consumption, with accessible primary amines decreasing from 48 ± 13 μmol g^–1^ in the starting material to 7 ± 2 μmol g^–1^ after conjugation (Table S3), confirming efficient FITC coupling and a degree of conjugation of *ca* *.* 86%. FITC conjugation also caused a marked ζ‐potential shift in Milli‐Q water from +26.2 ±  0.4 to −18.5 ± 1.9 mV, resulting from both amine depletion and partial interfacial FITC deprotonation at near‐neutral pH. DLS under the same conditions showed an increased hydrodynamic diameter from 48.6 ± 0.1 to 99.9 ± 2.1 nm after FITC functionalization (Figure 2e), consistent with minor aggregation. This was confirmed by TEM analysis, which revealed spherical nanoparticles with an average diameter (29.2 ± 7.0 nm) consistent with the starting material, but with a moderate aggregation tendency, although less pronounced than in the control NPs‐NoAm sample (compare Figure 2d with Figures 1e,g and S7c). These results establish a simple but effective protocol to modify the optical properties of the amine‐functionalized polymeric nanoparticles via amine‐isothiocyanate chemistry, featuring effective unreacted fluorophore removal and conjugation quantitative analysis. They also highlight the critical importance of surface charge control in preventing aggregation, as systematically demonstrated through comprehensive characterization.

The introduction of thermoresponsive properties to the NPs‐Am was achieved by reaction of the nanoparticle interfacial amines with the activated carboxylic groups of p(NIPAM_51_‐AANHS_3_) polymer (Figure 2f).

The thermoresponsive copolymer p(NIPAM_51_‐AANHS_3_) was successfully synthesized via RAFT polymerization, achieving 85% monomer conversion (Section S2.7). ^1^H‐NMR characterization confirmed the target degree of polymerization (DP NIPAM = 51, DP AANHS = 3) and an estimated *M*_*n*_ of 6,500 g mol^−1^ (Figures S10 and S11). TD‐GPC analysis revealed narrow dispersity (*Ð* = 1.018 ± 0.003), with *M*_*n*_ = 9,865 ± 278 g mol^−1^ and *M*_*w*_ = 10,047 ± 286 g mol^−1^ (Figure S12), consistent with well‐controlled RAFT polymerization. UV–vis turbidimetry measurements (450 nm, 20°C–45°C in Milli‐Q water) revealed the thermoresponsive behavior of the polymer, exhibiting an LCST of 34°C ± 1°C and a transition slope factor of 0.55°C ± 0.02°C (from heating ramp, Figure S13). Although the heating‐cooling cycle was reversible, a measurable hysteresis was observed during cooling, consistent with a complex coil‐to‐globule transition involving four thermodynamically stable intermediate states [32]. The determined LCST aligns closely with reported values for analogous p(NIPAM) systems (31°C–34°C), further validating the findings [33, 34].

The conjugation of p(NIPAM_51_‐AANHS_3_) onto NPs‐Am was achieved by directly mixing the polymer and the nanoparticles in a 1:1 weight ratio in carbonate buffer (100 mM, pH 8.5) overnight (Section S2.8). Removal of unreacted p(NIPAM_51_‐AANHS_3_) was achieved by dialysis against Milli‐Q water for 48 h. The successful conjugation was verified through ^1^H‐NMR spectroscopy, which displayed the characteristic signal of the isopropyl groups of p(NIPAM) at around 3.90 ppm (Figure S14). Moreover, the depletion of amino groups as a consequence of their reaction with NHS moieties was checked by the Kaiser test, showing a reduced number of interfacial primary amines (4.3 ± 0.3 μmol g^–1^, Table S4), consistent with a degree of conjugation of ca. 91%. Consistent with the FITC‐tagged nanoparticles, the p(NIPAM_51_‐AANHS_3_)‐functionalized p(GMA_35_‐AEMA_2_‐HPMA_101_‐EGDMA_16_) nanoparticles (p(NIPAM)@NPs‐Am) exhibited a significant ζ‐potential reduction in Milli‐Q water, shifting from +26.2 ± 0.4 to –36.1 ± 1.3 mV. This change reflects both amine group depletion and conversion of unreacted AANHS monomers to deprotonated carboxylic acids at nearly neutral pH. DLS measurements in Milli‐Q water showed an increased hydrodynamic diameter from 48.6 ± 0.1 to 86.8 ±  0.5 nm, consistent with partial nanoparticle aggregation (Figure 2g). TEM analysis confirmed this result, demonstrating preservation of spherical morphology with unchanged core nanoparticle size (30.0 ± 5.8 nm), though with moderate aggregation likely due to suboptimal surface charge (Figures 2h and S7d).

The thermoresponsive behavior of p(NIPAM)‐coated nanoparticles was investigated by variable‐temperature DLS from 20°C to 56°C in Milli‐Q water. The nanoparticles exhibited a characteristic sigmoidal response, with hydrodynamic diameter decreasing from 78.0 ± 1.0 nm at 20°C to 70.6 ± 0.4 nm at 56°C (9.6% ± 0.6% reduction; Figures 2i and S15). Notably, the LCST of p(NIPAM)@NPs‐Am increased to 41°C ± 1°C (Figure S15c) compared to the unconjugated p(NIPAM_51_‐AANHS_3_) (34°C ± 1°C), suggesting altered hydration dynamics upon surface immobilization. The thermoresponsiveness was fully reversible with no measurable hysteresis, as demonstrated by the overlapping heating and cooling cycles (Figure 2i). In contrast, control experiments with pristine NPs‐Am showed no temperature‐dependent size variation (Figures 2i and S15b), demonstrating that the thermoresponsive behavior originated specifically from the p(NIPAM) coating. The observed LCST increase (from 34°C to 41°C) reflects constrained dehydration behavior of surface‐grafted p(NIPAM_51_‐AANHS_3_) in its multipoint attachment conformation. Compared to free p(NIPAM_51_‐AANHS_3_) in water solution, the multipoint‐immobilized polymer chains exhibit: (1) reduced chain mobility limiting dehydration and (2) coil‐to‐globule conformational changes confined primarily to loop regions between attachment points, rather than across the entire polymer chain [35, 36]. This restricted conformational freedom explains both the higher transition temperature and the attenuated phase separation magnitude relative to linear p(NIPAM) in solution.

Given that carbonate buffer (100 mM, pH 8.5) constitutes the operative medium for both conjugation reactions, we evaluated the temporal stability of surface amine groups under these conditions. Prolonged exposure of NPs‐Am to carbonate buffer for 48 h resulted in a near‐complete loss of accessible amine groups, as indicated by the Kaiser test and confirmed by ζ‐potential inversion (Table S5). DLS analysis showed that well‐defined nanoparticles were preserved throughout, with no evidence of aggregation or structural collapse, consistent with aminolysis occurring selectively at the nanoparticle surface (Section S3) [37, 38]. Critically, FITC labeling and p(NIPAM_51_‐AANHS_3_) conjugation were both carried out overnight, a timescale at which amine functionality is preserved and reactivity toward NHS‐activated esters is favored over competing hydrolytic processes. The functional outcomes, namely fluorescent labeling and the acquisition of thermoresponsive behavior, corroborate that nanoparticle integrity and surface reactivity are fully maintained under the operative conjugation conditions.

From a general perspective, we have developed a robust conjugation and purification protocol for functionalizing NPs‐Am with tailored polymers, enabling precise modification of their physicochemical properties while maintaining quantitative control over conjugation efficiency. As a proof of concept, we demonstrated that p(NIPAM_51_‐AANHS_3_) conjugation confers reversible thermoresponsive behavior, with measurable temperature‐dependent size changes. This versatile platform can be readily extended to other functional polymers, offering broad opportunities for engineering stimuli‐responsive nanocarriers with customized properties and functions.

### 2.3 Self‐Assembly of Amine‐Functionalized Block Copolymer Nanoparticles Into Crosslinked Colloidosomes

The amphiphilic nature of NPs‐Am and their interfacial reactivity, which is due to the presence of readily available amines, make them ideal candidates for the self‐assembly and chemical crosslinking of micrometer‐sized colloidosomes.

**FIGURE 3:**
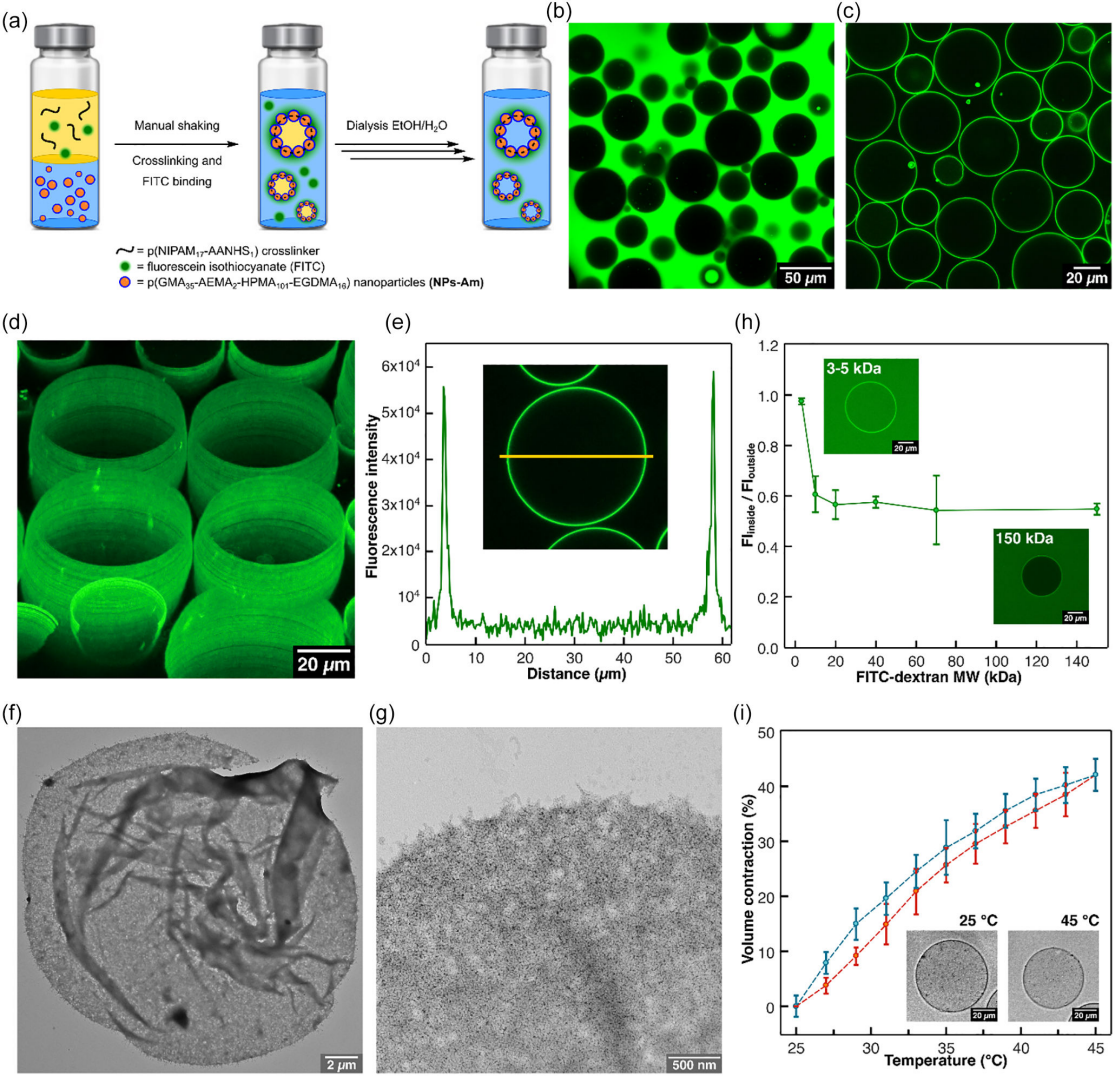
(a) Scheme showing the general procedure for the preparation of o/w Pickering emulsions, their tagging with FITC, chemical crosslinking with p(NIPAM_17_‐AANHS_1_) polymer, and subsequent transfer to water to obtain colloidosomes. (b) Confocal fluorescent microscopy image showing a population of crosslinked o/w microdroplets stabilized by NPs‐Am. Green fluorescence is due to FITC present in the external aqueous phase. (c) Confocal fluorescent microscopy image of colloidosomes in water. (d) 3D Z‐stack micrograph of colloidosomes recorded by confocal fluorescent microscopy. (e) A fluorescent intensity line profile referring to the yellow area on the corresponding confocal microscopy image. (f) Representative TEM image of a single dried and collapsed colloidosome. (g) High‐magnification TEM image of the membrane in (f), revealing its nanostructure composed of crosslinked polymeric nanoparticles. (h) Graph describing the ratio of fluorescence intensity inside (FI_inside_) to outside the colloidosome membrane (FI_outside_) as a function of the molecular weight (MW) of FITC‐dextran. Microscopy images show colloidosomes where FITC‐dextran fully permeated the membrane (top) and remained in the external environment (bottom). (i) Graph showing the temperature‐dependent reversible contraction (volume %) of colloidosomes crosslinked with p(NIPAM_17_‐AANHS_1_) polymer (heating cycle, red plot; cooling cycle, light blue plot). The brightfield microscopy images show the same colloidosome at 25°C and at 45°C. In (h) and (i) error bars indicate standard deviation on three consecutive measurements performed on the same sample.

Colloidosomes were prepared via the Pickering emulsion method [39]. Briefly, 200 μL of a NPs‐Am dispersion (5 mg mL^−1^ in 100 mM carbonate buffer, pH 8.5) was layered with 200 μL of 2‐ethyl‐1‐hexanol as the oil phase (Figure 3a, Section S4). To simultaneously crosslink the colloidosomes for subsequent transfer into an aqueous phase and fluorescently label them for confocal microscopy, a mixture of low molecular weight p(NIPAM_17_‐AANHS_1_) crosslinker synthesized via free‐radical polymerization (0.1 mg in 20 μL chloroform, Section S2.9, Figures S16 and S17) and FITC (5 μL of 1 mg mL^−1^ in DMSO) was added to the oil phase.

The system was vigorously shaken by hand to form an oil‐in‐water (o/w) Pickering emulsion, where the amphiphilic nanoparticles self‐assembled at the oil–water interface, stabilizing microdroplets with an average diameter of 26.4 ± 11.9 μm (Figures 3b and S19a). The o/w nature of the emulsions was confirmed by confocal microscopy, which showed that water‐soluble FITC was confined to the external aqueous phase (Figure 3b). This is consistent with the hydrophilic p(GMA)‐based nanoparticle corona produced by RAFT‐PISA, which favors o/w emulsion stabilization [40].

The crosslinker and FITC were then allowed to react with the nanoparticle amine groups for 48 h. Subsequently, the colloidosomes were transferred to water via sequential dialysis, first against 70% ethanol (EtOH) for 5 h, then 30% EtOH for 3 h, and finally against Milli‐Q water overnight. The resulting crosslinked and fluorescently labeled colloidosomes in water exhibited a hollow spherical structure (Figure 3c–e) with an average diameter of 41.5 ± 10.7 μm (Figure S19b). Additionally, TEM images confirmed the hollow morphology, revealing collapsed, dried structures composed of densely packed, crosslinked nanoparticles (Figure 3f,g).

To study the permeability properties of crosslinked colloidosome membranes, water‐transferred colloidosomes were incubated with FITC‐labeled dextrans of varying molecular weights (3–150 kDa). Using fluorescence confocal microscopy, the ratio of fluorescence intensity inside (FI_inside_) to outside (FI_outside_) the colloidosome membrane was evaluated. The results indicated that the MWCO lies between 3 and 10 kDa, with smaller dextrans permeating the membrane while larger molecules remained excluded (Figure 3h, Section S4.1). These findings demonstrate that colloidosomes exhibit selective permeability based on membrane MWCO, enabling the design of carriers with tailored permeability for controlled cargo retention or release in drug delivery applications.

To characterize the thermoresponsive behavior of crosslinked colloidosomes, the LCST of p(NIPAM_17_‐AANHS_1_) crosslinker was first determined, showing an LCST of 31°C ± 1°C (from heating ramp) and a transition slope factor of 1.44°C ± 0.02°C, consistent with typical phase transition behavior of p(NIPAM)‐based polymers (Figure S18). Colloidosomes assembled with NPs‐Am and crosslinked with p(NIPAM_17_‐AANHS_1_) were subjected to temperature‐dependent imaging to investigate membrane contractility.

Increasing the temperature from 25°C to 45°C with 2°C increments induced pronounced membrane contraction, with a maximum volume reduction of 42.1% ± 2.9% at 45°C. This contractility confirmed that the crosslinked membrane responds dynamically to thermal stimuli through integration of the thermoresponsive polymer. The thermoresponsive behavior was fully reversible with no measurable hysteresis, as demonstrated by overlapping heating and cooling cycles (Figure 3i, Section S4.2). These results align with the thermoresponsive properties of p(NIPAM)‐coated nanoparticles previously characterized (Figure 2i).

These results demonstrate the versatility of our amine‐functionalized block‐copolymer nanoparticles as modular building blocks for constructing novel microcompartmentalized systems, that could be exploited for applications in drug delivery, microreactor technology, and protocell engineering. The rational design of functional polymeric nanoparticles enabled by RAFT‐PISA, combined with the precise characterization methods reported here, opens new avenues for engineering innovative functional microstructures with tunable properties.

## 3 Conclusions

This study bridges the gap between molecular‐scale design and macroscopic functionality by establishing a robust platform for the synthesis and precise engineering of amine‐functionalized polymeric nanoparticles via RAFT‐PISA. The incorporation of AEMA into the macroCTA not only enabled the formation of stable, monodisperse nanoparticles, but also provided a versatile handle for postpolymerization modifications, addressing the longstanding challenge of correlating molecular design with functional performance. Rather than demonstrating amine incorporation as a synthetic end point, this work establishes a quantitative structure‐reactivity framework that links molecular‐scale amine accessibility to macroscopic functional outcomes. Quantitative characterization revealed the accessibility of interfacial amines, facilitating efficient conjugation with FITC (86% yield) for optical tracking and thermoresponsive p(NIPAM) (91% efficiency), the latter exhibiting a tunable LCST transition at 41°C due to constrained polymer dynamics. Furthermore, the nanoparticle amphiphilic nature and reactive amines allowed their hierarchical assembly into crosslinked colloidosomes exhibiting temperature‐induced contractility (up to 40% of initial volume) and selective permeability (MWCO between 3 and 10 kDa). The consistency of these outcomes across two chemically distinct conjugation pathways and a hierarchical assembly strategy positions this work not as a single synthetic contribution, but as a platform methodology with broad applicability.

From a broader perspective, this work establishes a replicable approach for engineering functional polymeric nanoparticles with quantitative precision, shifting the field focus from morphological design to chemical programmability and postassembly reactivity. In drug delivery, our quantitative conjugation metrics enable control over ligand density for targeted uptake and payload capacity for optimized therapeutic efficacy. For diagnostic systems, the well‐defined fluorescent labeling ensures accurate tracking and quantification in complex biological environments. In nanoreactor design, the characterized interfacial chemistry permits rational optimization of catalytic sites and compartmentalized reactions. The colloidosome platform extends these capabilities to microbioreactor technologies, where semipermeable, stimuli‐responsive compartments could mimic cellular environments for biocatalysis or synthetic biology. Additionally, the possibility to tailor membrane permeability and surface reactivity of colloidosomes based on nanoparticle building block design opens new avenues for protocell engineering, enabling the bottom‐up construction of artificial cells with programable communication and metabolic behaviors.

Future research could explore polymers responsive to multiple stimuli (e.g., pH, redox) or bioactive ligands (e.g., peptides, sugars) to further expand the versatility of this platform. By establishing a quantitative link between interfacial chemistry and hierarchical functionality, this approach advances the field from empirical optimization toward true molecular engineering of polymeric nanomaterials, a direction with transformative implications across biomedical and bioengineering applications.

## Supporting Information

Additional supporting information can be found online in the Supporting Information section.

## Author Contributions

**A. S** **.**, **F. B** **.**, **P. T.**, and **P. G.** conceived and planned the experiments. **A. S.**, **B. R.**, **S. V.**, and **M. S.** carried out the experiments. **A. S.**, **B. R.**, **S. V.**, **M. S.**, **F. B.**, and **P. G.** undertook data analysis, discussed the results and contributed to the interpretation of the results. **A. S.**, **F. B**., **P. T.**, and **P. G.** wrote the final manuscript. All authors provided critical feedback toward the final manuscript.

## Funding

This study was supported by the European Union Next Generation EU (2022285HC5_002, P2022BLNCS and CN00000041) and European Research Council (101039578).

## Conflicts of Interest

The authors declare no conflicts of interest.

## Supporting information

Supplementary Material

## Data Availability

The data that support the findings of this study are available from the corresponding author upon reasonable request.
