# Supplementary material for: Quantifying and Leveraging Interfacial Amine Reactivity in Block Copolymer Nanoparticles for Advanced Material Design
Source: Small Sci. 2026 May 15;6(5):e70295. doi: 10.1002/smsc.70295 (PMC13179043; doi:10.1002/smsc.70295)
Supplement: Supplementary file 1 — Supplementary Material [file SMSC-6-e70295-s001.pdf]

Supporting Information

Quantifying and Leveraging Interfacial Amine Reactivity in Block Copolymer Nanoparticles for Advanced Material Design

Aharon Steffè[1], Beatrice Rosetti[1], Stefano Valente[1], Maria Sbacchi[1,2], Federica Battistin[1], Paolo Tecilla*[1], Pierangelo Gobbo*[1,2]

Affiliations:

1. Department of Chemical and Pharmaceutical Sciences, University of Trieste; Via L. Giorgieri 1, 34127, Trieste (Italy).
2. National Interuniversity Consortium of Materials Science and Technology, Unit of Trieste; Via G. Giusti 9, 50121, Firenze (Italy).

Table of content

1. Materials and Methods ... 3

2. Synthesis and characterization of polymers and nanoparticles ... 7

2.1 Poly(glycerol monomethacrylate-*co*-2-aminoethyl methacrylate) macromolecular Chain Transfer Agent, macroCTA-Am ... 7

2.2 Evaluation of surface reactive amino groups on polymers and nanoparticles ... 8

2.3 Poly(glycerol monomethacrylate) macroCTA, macroCTA-NoAm[2] ... 9

2.4 Poly(glycerol monomethacrylate-*co*-2-aminoethyl methacrylate-*co*-2-hydroxypropyl methacrylate-*co*-ethylene glycol dimethacrylate) nanoparticles, NPs-Am 10

2.5 Poly(glycerol monomethacrylate-*co*-2-hydroxypropyl methacrylate-*co*-ethylene glycol dimethacrylate) nanoparticles, NPs-NoAm ... 13

2.6 Functionalization of NPs-Am with FITC ... 13

2.7 Poly(*N*-isopropylacrylamide-*co*-*N*-acryloxysuccinimide), p($NIPAM_{51}$-$AANHS_3$) ... 14

2.8 Functionalization of p(GMA_{35}-$AEMA_2$-$HPMA_{101}$-$EGDMA_{16}$) nanoparticles with p($NIPAM_{51}$-$AANHS_3$) polymer, p(NIPAM)@NPs-Am ... 17

2.9 Poly(*N*-isopropylacrylamide-*co*-*N*-acryloxysuccinimide), p($NIPAM_{17}$-$AANHS_1$) ... 18

3. Stability studies of NPs-Am in carbonate buffer for 48 hrs ... 20

4. Preparation of Pickering emulsions and stable w/w colloidosomes ... 20

4.1 Permeability studies of crosslinked w/w colloidosomes ... 21

4.2 Contractile temperature-dependent behaviour of crosslinked w/w colloidosomes 21

1. Materials and Methods

All reagents were used as received unless otherwise stated. Glycerol monomethacrylate (GMA; ≤100%) was purchased from Polysciences. 2-hydroxypropyl methacrylate (HPMA; ≥97%), 2-aminoethyl methacrylate hydrochloride (AEMA; 90%), *N*-Isopropylacrylamide (NIPAM; 97%), 2-cyano-2-propyl dithiobenzoate (CPDB), 4-cyano-4-(((propylthio)carbonothioyl)thio)pentanoic acid (95%), 4,4'-azobis(4-cyanopentanoic acid) (ACVA; ≥98%), α,α′-Azoisobutyronitrile (AIBN; ≥98.0%), fluorescein isothiocyanate (FITC; ≥90%), deuterium oxide (D_2O; 99.9%), deuterated methanol (CD_3OD; ≥99.8%), deuterated chloroform ($CDCl_3$; ≥99.8%), 2-ethyl-1-hexanol (≥99.6%), fluorescein isothiocyanate-dextran (FITC-dextran) were purchased from Sigma-Aldrich. Ethylene glycol dimethacrylate (EGDMA; 98%) was purchased from Sigma-Aldrich and purified through aluminium oxide 90 active basic (0.063-0.200 mm) and silica gel 60 (0.063-0.200 mm) plugs to remove monomethyl ether hydroquinone (MEHQ) inhibitor before use. *N*-Acryloxysuccinimide (AANHS; 99%) and Spectrum™ Labs Spectra/Por™ dialysis membranes (MWCO 3500 and 14000) were purchased from Thermo Fisher Scientific. All solvents were of analytical grade and purchased from Sigma-Aldrich.

The NMR spectra were recorded on a Varian 400 MHz spectrometer (^{1}H: 400 MHz, ^{13}C: 100.5 MHz). The chemical shift (δ) for ^{1}H and ^{13}C is given in ppm relative to signals of the residual solvents ($CHCl_3$ δ at 7.26 ppm, HDO δ at 4.79 ppm, and CH_3OH δ at 3.31 ppm).

The monomer percentage of conversion and the degree of polymerization (DP) of synthesized polymers and nanoparticles were calculated from the ^{1}H-NMR spectra of the reaction mixture solution taken before (t_0) and at the end (t_f) of the polymerization reaction. *N,N*-dimethylformamide (DMF) (100 µL, 1.29 mmol) was used as an NMR internal standard for calculations. For p(GMA)-based polymers and nanoparticles, the monomer conversion and DP were calculated by comparing the integrated monomer vinyl proton signals at 5.61–5.67 ppm with the DMF singlet at 7.97 ppm (in CD_3OD) or 7.92 ppm (in D_2O). For p(NIPAM-AANHS)-based polymers, the monomer conversion and DP were calculated by comparing the integrated monomer proton signals at 4.00–4.15 ppm with the DMF singlet at 8.02 ppm (in $CDCl_3$).

Dynamic light scattering (DLS) measurements were performed using a Zetasizer Nano ZS instrument (Malvern Panalytical). Intensity-average hydrodynamic diameter and ζ-potential analysis of nanoparticles (0.2 mg mL^{-1}) in pre-filtered Milli-Q water (0.22 µm nylon syringe filter) were performed in backscattering mode at 173° scattering angle, with controlled temperature at 25 °C in folded capillary electrode cells DTS1070 (Malvern Panalytical) and with three consecutive measurements for each sample. For temperature-dependent DLS measurements, intensity-average hydrodynamic diameters of nanoparticles (0.2 mg mL^{-1}) in pre-filtered Milli-Q water (0.22

μm nylon syringe filter) were determined in a temperature range from 20 to 56 °C with three consecutive measurements every 2 °C and an equilibration time of 120 sec in 1 cm path length quartz cuvettes. For pH-dependent DLS measurements, intensity-average hydrodynamic diameter and ζ-potential of nanoparticles (0.2 mg mL^{-1}) in pre-filtered 1 mM KCl solution (0.22 μm nylon syringe filter) were determined in a pH range from 4 to 12 (pH was adjusted using HCl and NaOH 0.1 M) with three consecutive measurements every 1 unit of pH and an equilibration time of 120 sec in folded capillary electrode cells DTS1070 (Malvern Panalytical).

UV-Vis spectroscopy was acquired with UV2450 spectrophotometer (Shimadzu). All measurements were performed acquiring the spectrum from 200 to 800 nm wavelength against Milli-Q water as reference for blank subtraction. Samples were prepared by dissolving a precise amount of polymers or nanoparticles in Milli-Q water to a final concentration of 1 mg mL^{-1} in 1 cm path length quartz cuvettes, unless otherwise stated.

Excitation and emission spectra were recorded using an Edinburgh Instruments FS5 spectrofluorometer equipped with a Xenon arc lamp as the excitation source and a photomultiplier tube (PMT) detector. Measurements of FITC-tagged nanoparticles in Milli-Q water (0.3 mg mL^{-1}) were conducted at room temperature in a 1 cm path length quartz cuvette. The excitation spectrum was recorded by fixing the emission wavelength at 550 nm, while scanning the excitation wavelengths from 200 nm to 540 nm. The emission spectrum was acquired by exciting the sample at 480 nm and scanning the emission wavelengths from 485 nm to 800 nm. Steps for both excitation and emission were set to 1 nm, and integration time (dwell time) was set to 0.2 sec. All spectra were corrected for variations in lamp output and detector sensitivity using the instrument's automated spectral correction files, ensuring accurate representation of sample fluorescence.

The thermoresponsive properties of polymers in solution were characterized by monitoring the transmittance of polymer solutions using an Agilent Cary 3500 UV-Vis spectrophotometer in 1 cm path length quartz cuvettes. The instrument is equipped with long-life Xenon flashlamp source (250 Hz), a wavelength range of 190-1100 nm, a limiting resolution of 0.1 nm, and a silicon photodiode detector. Samples were prepared at a concentration of 1 mg mL^{-1} in Milli-Q water and were measured against a reference sample, blanks were automatically subtracted. Kinetic experiments were carried out by monitoring the absorbance at 450 nm of stirred polymer solutions (600 rpm) in a temperature range from 20 to 45 °C with a rate of 0.5 °C min^{-1}, a 0.5 °C data interval, a spectral bandwidth of 1.00 nm, an averaging time of 1.00 sec, and a hold time of 0.10 min. The temperature was accurately measured using a probe directly inserted into the analyte cuvette. The absorbance was converted into transmittance % (T%) and the lower critical solution temperature

(LCST) was determined by fitting experimental data with the Boltzmann equation. The general form of the Boltzmann sigmoidal function is:

$$y = \frac{A_1 - A_2}{1 + e^{(x-x_0)/dx}} + A_2$$

where A_1 and A_2 represent the upper and lower asymptotes (reflecting the baseline values before and after the transition), x_0 is the inflection point (corresponding to the midpoint of the transition, the LCST), and *dx* determines the slope factor which indicates the sharpness of the transition. This approach provides a precise, quantitative way to extract LCST information (*i.e.*, transition temperature and sharpness) from experimental data.

Polymers were characterized using tetra-detection gel permeation chromatography (TD-GPC) with an Omnisec RESOLVE/REVEAL system (Malvern Panalytical) equipped with refractive index (RI), UV-Vis, right angle light scattering (RALS)/low angle light scattering (LALS), and differential viscometer detectors. All data were collected and processed using Omnisec v12 software. The multi-detector system was calibrated using PolyCALTM Pullulan (narrow distribution – calibration, 107 K) and dextran (broad distribution – verification, 68 K) standards (Malvern Panalytical). All the mobile phases used were pre-filtered through a bottle vacuum filter funnel with a 0.22 μm polyethersulfone membrane (Steritop®, Merck). For the chromatographic separation of synthetic polymers, two cationic columns (TSKgel G6000PWXL-CP + G3000PWXL-CP, Tosoh Bioscience) were employed, the mobile phase used was a 0.1 M $NaNO_3$ aqueous solution at pH 2.6, acidified with acetic acid (*ca.* 0.5% v/v), the flow rate was 0.5 mL min^{-1}. The temperature of column and detector oven was 20 °C, whereas the autosampler was kept at 4 °C. P($NIPAM_{51}$-$AANHS_3$) samples were prepared at a concentration of *ca.* 3 mg mL^{-1} in the eluent and filtered through 0.45 μm regenerated cellulose syringe filters. The *dn*/*dC* used was experimentally determined and corresponded to 0.187 ± 0.011 mL g^{-1}. Results were the average of three separate runs, the error was calculated using standard deviation.

The determination of molecular weight and molecular weight distribution by TD-GPC of the GMA-based polymers proved unfeasible in both aqueous and DMF systems due to strong interactions between the polymer and the stationary phase. These interactions, likely resulting from the polymer amphiphilic character and the high number of hydroxy groups, prevented accurate size-exclusion separation and meaningful data acquisition. Moreover, the same polymers were insoluble in tetrahydrofuran (THF), another standard solvent for GPC.

Optical and fluorescence microscopy was performed on a Axio Observer 7 (Zeiss) microscope at 20× and 40× magnification. The microscope was equipped with an Orca Flash 4.0 V3 CMOS camera (Hamamatsu) and a motorized XYZ sample stage. The brightfield images were obtained using a transmitted light LED lamp.

Confocal laser scanning microscopy images were acquired on a FV3000 (Olympus) confocal laser scanning microscope (CLSM). The system was equipped with 5 excitation laser lines: 375, 405, 488, 561, and 640 nm, a galvanometric scanning head, and 4 spectral detectors, allowing for simultaneous imaging of up to 4 fluorescence channels. Brightfield images were acquired using a scanning laser line and a transmitted light detector. The following objectives were used: UPLXAPO 20×/0.8 NA (Olympus), and an oil objective UPLXAPO 60×/1.42 NA (Olympus). Imaging of FITC-tagged Pickering emulsions and colloidosomes was performed using an excitation wavelength of 488 nm and an emission wavelength range of 500-550 nm. Analysis of Pickering emulsions and colloidosomes' size distribution was performed by threshold-based segmentation with the software suite Zen (Zeiss).

Transmission electron microscopy (TEM) images were acquired with an EM 208 Electron microscope (Philips) at 100 kV and equipped with a 11 MP digital camera (Olympus Quemesa). The samples were prepared by depositing aqueous dispersions of nanoparticles (0.2 mg mL^{-1}, 10 μL) onto TEM grids (CF200-Cu; carbon support film 200 mesh, copper). Samples were let deposit for 2 min and then blotted with filter paper to remove excess sample solution. For the staining, uranyl acetate (0.75 mg mL^{-1}) aqueous solution (9 μL) was dropped on grids for 30 sec and then cautiously blotted with filter paper to avoid excessive staining. The grids were dried at room temperature under a chemical hood. Images were analyzed with ImageJ (National Institutes of Health) software to obtain nanoparticles size distribution (a minimum of 200 nanoparticles *per* batch were considered).

2. Synthesis and characterization of polymers and nanoparticles

2.1 Poly(glycerol monomethacrylate-*co*-2-aminoethyl methacrylate) macromolecular Chain Transfer Agent, macroCTA-Am

CPDB RAFT agent (23.14 mg, 0.10 mmol), GMA monomer (1.00 g, 6.24 mmol) (target GMA DP = 47), AEMA monomer (hydrochloric acid salt, 51.93 mg, 0.31 mmol, target AEMA DP = 3), and ACVA initiator (5.86 mg, 0.02 mmol) (CPDB/ACVA ratio = 5.0) were weighted, dissolved in ethanol (1.5 mL), and transferred into a Schlenk tube equipped with a magnetic stirring bar. DMF (100 µL, 1.29 mmol) was added into the flask and used as NMR internal standard for monomer conversion percentage and DP calculations. The Schlenk tube was sealed, and the resulting red solution underwent at least five freeze-pump-thaw cycles with liquid nitrogen and, at the end, insertion of argon to remove the oxygen. Then, the reaction was immersed in an oil bath set at 70 °C for 85 min and the polymerization was successively quenched by immersion in liquid nitrogen followed by exposure to air. Methanol (3 mL) was added to the reaction mixture to dissolve the polymer, followed by precipitation into twenty-fold excess of ice-cold dichloromethane (CH_2Cl_2). The crude macroCTA polymer was precipitated three times with CH_2Cl_2, evaporated under vacuum, dissolved in water, and freeze-dried for 48 hrs to yield a pink powder (375.00 mg, 38%). ^{1}H-NMR analysis of the purified macroCTA indicated a monomers conversion of 55%, a GMA DP of 35, an AEMA DP of 2, and an estimated mean molecular weight (M_n) of 6,022 g mol^{-1}.

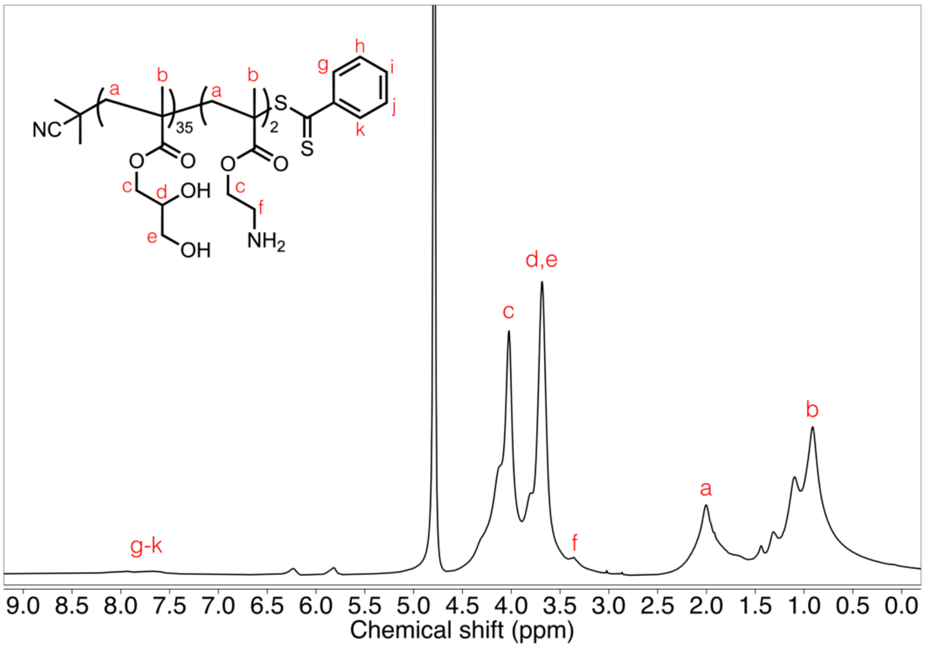

Figure S1. ^{1}H-NMR (400 MHz, D_2O) spectrum of macroCTA-Am in D_2O referenced against the peak at 4.79 ppm of HDO.

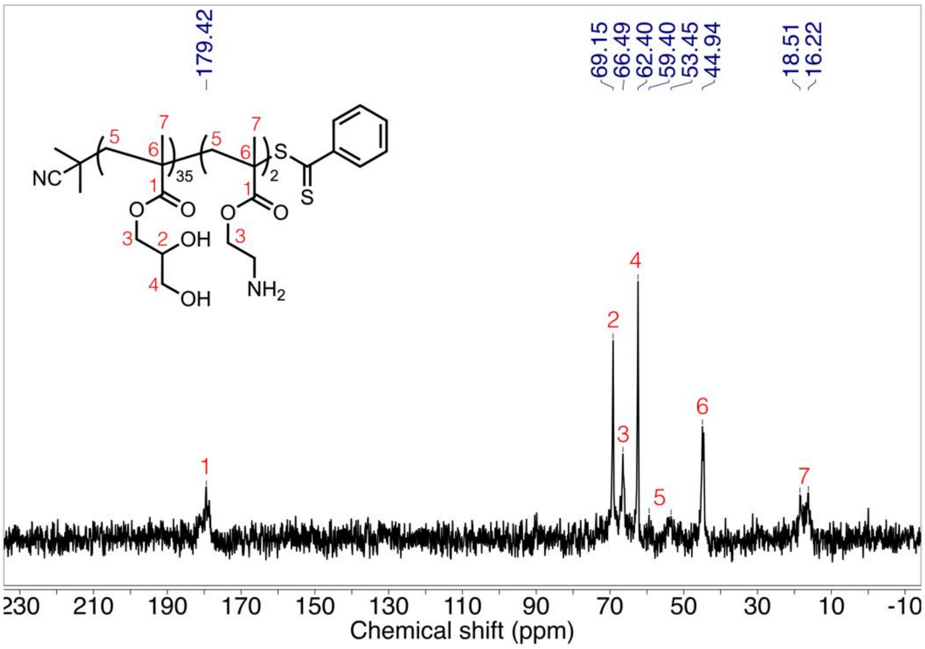

Figure S2. ^{13}C-NMR (100.5 MHz, D_2O) spectrum of macroCTA-Am in D_2O.

2.2 Evaluation of surface reactive amino groups on polymers and nanoparticles

The Kaiser test was performed using a commercially available kit (60017, Sigma-Aldrich) following manufacturer's instructions. Briefly, 1 mg of macroCTA was mixed with a total of 250 μL of three different kit reagents (phenol, potassium cyanide, and ninhydrin solutions) and the reaction mixture was let to react at 120 °C for 10 min and at room temperature for 10 min or 24 hrs. Finally, the solution was diluted to 3 mL with 60% EtOH in water prior to the UV-Vis spectroscopy analysis with UV2450 spectrophotometer (Shimadzu) instrument. All measurements were performed acquiring the spectrum from 200 to 800 nm against a reference (a mixture solution of kit reagents without the sample) for blank subtraction and analysed at 570 nm where the Ruhemann's purple compound shows its absorbance band (ε = 15000 M^{-1} cm^{-1}).[1] The same experimental procedure was used for all samples, with the exception of nanoparticles. For those, the absorbance of nanoparticles with no amino groups p(GMA_{35}-$HPMA_{102}$-$EGDMA_{16}$) (NPs-NoAm) was used as reference for blank subtraction to remove scattering contributions. For convenience, all results for the different systems analyzed in this work are summarized in Tables S1-S4 below.

Table S1. Quantification of surface reactive amino groups of macroCTA compounds.

Sample	Reaction parameters	Primary amines (μmol g^{-1})
macroCTA-NoAm	120 °C, 10 min	–1 ± 1
macroCTA-Am	120 °C, 10 min	80 ± 9
macroCTA-Am	r.T., 10 min	43 ± 2
macroCTA-Am	r.T., 24 hrs	56 ± 10

Table S2. Quantification of surface reactive amino groups of NPs-Am.

Sample	Reaction parameters	Primary amines (μmol g^{-1})
NPs-Am	120 °C, 10 min	48 ± 13
NPs-Am	r.T., 10 min	25 ± 1
NPs-Am	r.T., 24 hrs	27 ± 2

Table S3. Quantification of the surface reactive amino groups of FITC@NPs-Am.

Sample	Reaction parameters	Primary amines (μmol g^{-1})
FITC@NPs-Am	120 °C, 10 min	7 ± 2

Table S4. Quantification of surface reactive amino groups of PNIPAM@NPs-Am.

Sample	Reaction parameters	Primary amines (μmol g^{-1})
p($NIPAM_{51}$-$AANHS_3$)	120 °C, 10 min	0.3 ± 0.8
p(NIPAM)@NPs-Am	120 °C, 10 min	4.3 ± 0.3

2.3 Poly(glycerol monomethacrylate) macroCTA, macroCTA-NoAm[2]

CPDB RAFT agent (23.14 mg, 0.10 mmol), GMA monomer (1.00 g, 6.24 mmol) (target GMA DP = 47), and ACVA initiator (5.86 mg, 0.02 mmol) (CPDB/ACVA ratio = 5.0) were weighted, dissolved in ethanol (1.5 mL), and transferred into a Schlenk tube equipped with a magnetic stirring bar. DMF (100 μL, 1.29 mmol) was added into the flask and used as NMR internal standard for monomer conversion percentage and DP calculations. The Schlenk tube was sealed, and the resulting red solution underwent at least five freeze-pump-thaw cycles with liquid nitrogen and, at the end, insertion of argon to remove the oxygen. Then, the reaction was immersed in an oil bath set at 70 °C for 100 min and the polymerization was successively quenched by immersion in liquid nitrogen followed by exposure to air. Methanol (3 mL) was added to the reaction mixture to dissolve the polymer, followed by precipitation into twenty-fold excess of ice-cold CH_2Cl_2. The crude macroCTA polymer was precipitated three times with CH_2Cl_2, evaporated under vacuum, dissolved in water, and freeze-dried for 48 hrs to yield a pink powder (420.00 mg, 42%). ^{1}H-NMR analysis of the purified macroCTA indicated a monomer conversion of 56%, a GMA DP of 35, and an estimated M_n of 5810 g mol^{-1}.

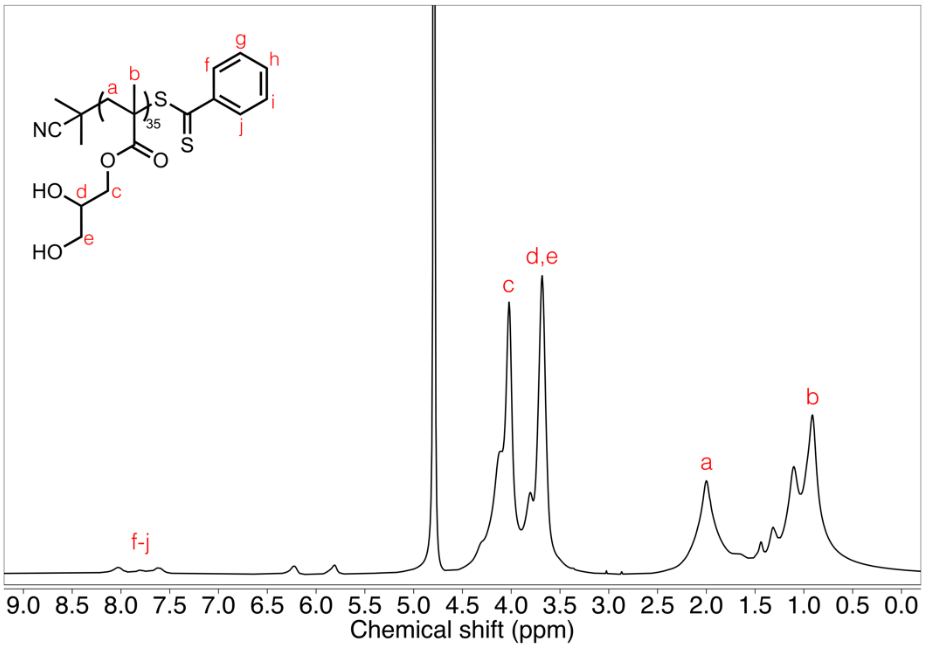

Figure S3. ^{1}H-NMR (400 MHz, D_2O) spectrum of macroCTA-NoAm in D_2O referenced against the peak at 4.79 ppm of HDO.

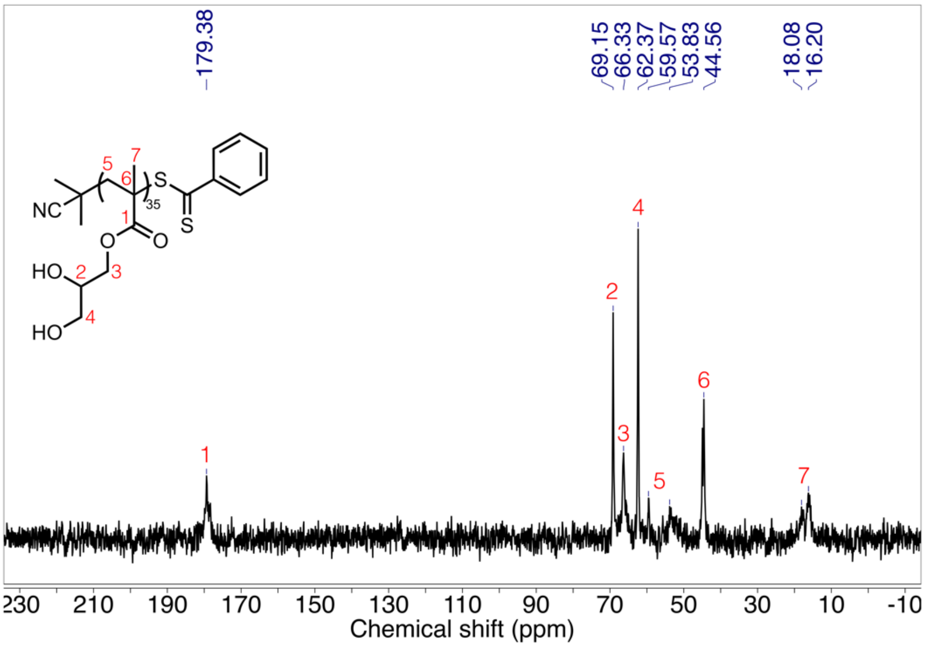

Figure S4. ^{13}C-NMR (100.5 MHz, D_2O) spectrum of macroCTA-NoAm in D_2O.

2.4 Poly(glycerol monomethacrylate-*co*-2-aminoethyl methacrylate-*co*-2-hydroxypropyl methacrylate-*co*-ethylene glycol dimethacrylate) nanoparticles, NPs-Am

macroCTA-Am (10.00 mg, 1.66 µmol), HPMA monomer (31.12 mg, 0.22 mmol) (target HPMA DP = 130), and ACVA initiator (0.15 mg, 0.55 µmol) (macroCTA/ACVA ratio = 3.0) were weighted, dissolved in water (4.8 mL, total solids content = 1 w/v%), and transferred into a Schlenk tube equipped with a magnetic stirring bar. DMF (100 µL, 1.29 mmol) was added into the flask and used as NMR internal standard for monomer conversion percentage and DP calculations. The Schlenk

tube was sealed, and the resulting solution underwent at least eight freeze-pump-thaw cycles with liquid nitrogen and, at the end, insertion of argon to remove the oxygen. Then, the reaction was immersed into an oil bath set at 70 °C for 24 hrs. After 5 hrs of HPMA polymerization, deoxygenated EGDMA monomer solution (6.58 mg, 0.03 mmol) (target EGDMA DP = 20) in water was introduced into the flask under an argon flux using a syringe. The polymerization was allowed to continue for the subsequent 19 hrs. At the end, the reaction was quenched by exposure to air and cooling to ambient temperature. The nanoparticles were purified by dialysis using Spectrum™ Labs Spectra/Por™ dialysis membrane (MWCO 12-14 kDa) for 48 hrs against Milli-Q water to remove any unreacted macroCTA and monomers. Finally, the product was freeze-dried for 48 hrs to obtain a dry white powder. ^{1}H-NMR analysis of the resulting turbid nanoparticles dispersion indicated a monomer conversion of 81%, a HPMA DP of 101, and an EGDMA DP of 16.

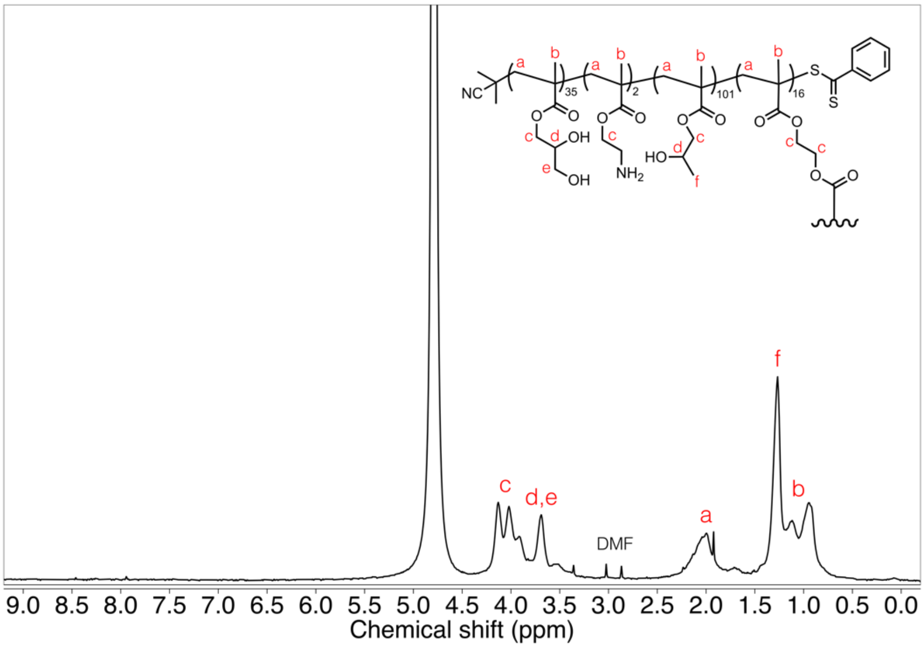

Figure S5. ^{1}H-NMR (400 MHz, D_2O) spectrum of NPs-Am in D_2O referenced against the peak at 4.79 ppm of HDO.

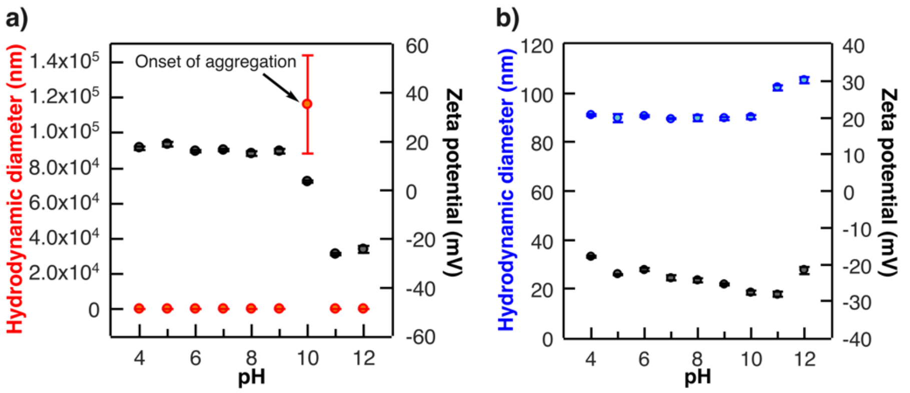

Figure S6. DLS intensity-average hydrodynamic diameter and ζ-potential as a function of solution pH for NPs-Am **(a)** and p(GMA_{35}-$HPMA_{102}$-$EGDMA_{16}$) nanoparticles (NPs-NoAm) **(b)** . Error bars indicate standard deviation on three consecutive measurements performed on the same sample. DLS measurements and ζ-potential were performed in 1 mM KCl solution with pH adjusted using HCl and NaOH 0.1 M. While NPs-NoAm remained stable across all tested pH values, NPs-Am exhibited flocculation at pH ~10, where deprotonation of interfacial amines caused the particles to reach their isoelectric point and lose electrostatic stabilization. This result is consistent with reported pKa values of similar systems.[3]

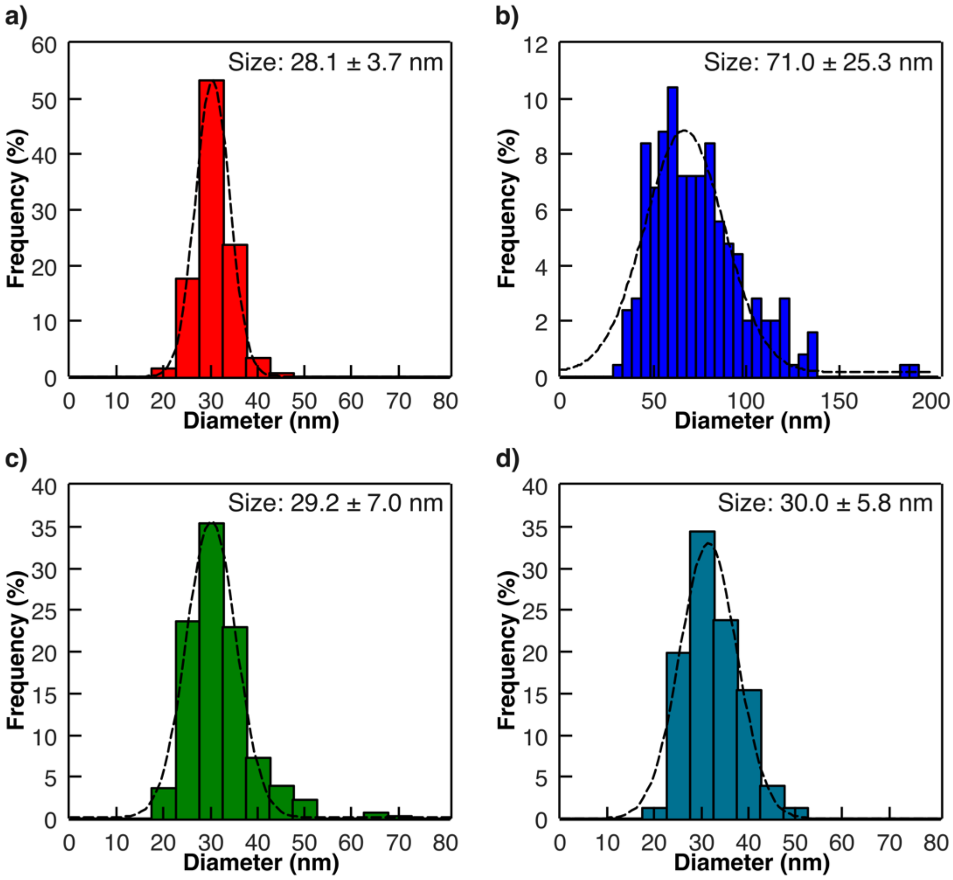

Figure S7. Plots showing nanoparticle size distributions determined from TEM images of **(a)** NPs-Am, **(b)** NPs-NoAm, **(c)** FITC-tagged p(GMA_{35}-$AEMA_2$-$HPMA_{101}$-$EGDMA_{16}$) (FITC@NPs-Am), and **(d)** p($NIPAM_{51}$-$AANHS_3$)-coated p(GMA_{35}-$AEMA_2$-$HPMA_{101}$-$EGDMA_{16}$) nanoparticles (p(NIPAM)@NPs-Am). The dashed black curves represent the Gaussian fitting of data.

2.5 Poly(glycerol monomethacrylate-*co*-2-hydroxypropyl methacrylate-*co*-ethylene glycol dimethacrylate) nanoparticles, NPs-NoAm

p(GMA_{35}) macroCTA (10.00 mg, 1.72 μmol), HPMA monomer (32.26 mg, 0.22 mmol) (target HPMA DP = 130), and ACVA initiator (0.16 mg, 0.57 mmol) (macroCTA/ACVA ratio = 3.0) were weighted, dissolved in water (5 mL, total solids content = 1 w/v%), and transferred into a Schlenk tube equipped with a magnetic stirring bar. DMF (100 μL, 1.29 mmol) was added into the flask and used as NMR internal standard for monomer conversion percentage and DP calculations. The Schlenk tube was sealed, and the resulting solution underwent at least eight freeze-pump-thaw cycles with liquid nitrogen and, at the end, insertion of argon to remove the oxygen. Then, the reaction was immersed into an oil bath set at 70 °C for 24 hrs. After 5 hrs of HPMA polymerization, deoxygenated EGDMA monomer solution (6.82 mg, 0.03 mmol) (target EGDMA DP = 20) in water was introduced into the flask under argon flux using a syringe. The polymerization was allowed to continue for the subsequent 19 hrs. At the end, the reaction was quenched by exposure to air and cooling to ambient temperature. The nanoparticles were purified by dialysis using Spectrum™ Labs Spectra/Por™ dialysis membrane (MWCO 12-14 kDa) for 48 hrs against Milli-Q water to remove any unreacted macroCTA and monomers. Finally, the product was freeze-dried for 48 hrs to obtain a dry white powder. ^{1}H-NMR analysis of the resulting turbid nanoparticles dispersion indicated a monomer conversion of 79%, a HPMA DP of 102, and an EGDMA DP of 16.

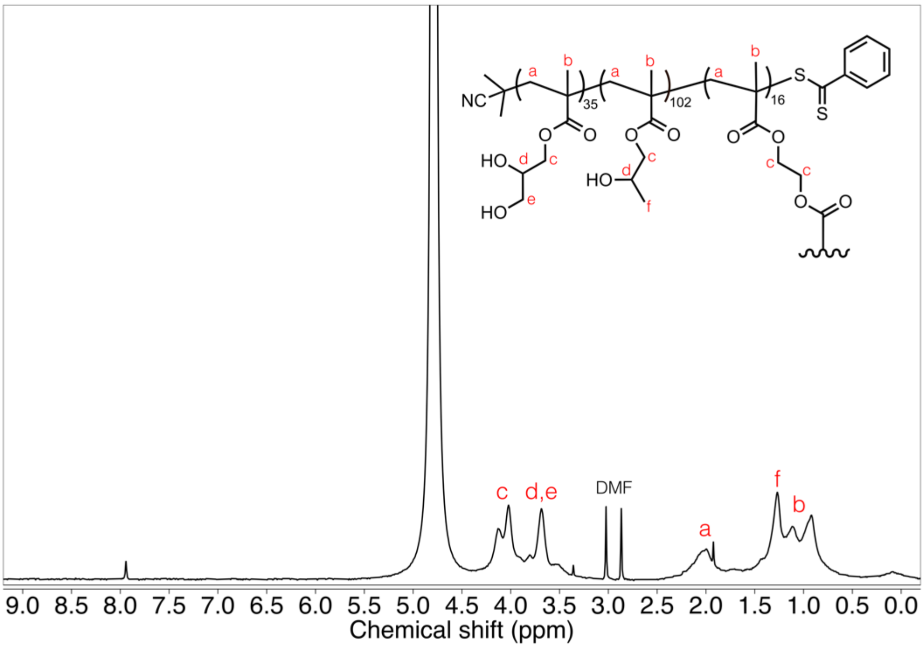

Figure S8. ^{1}H-NMR (400 MHz, D_2O) spectrum of NPs-NoAm in D_2O referenced against the peak at 4.79 ppm of HDO.

2.6 Functionalization of NPs-Am with FITC

In a vial, 9.9 mg of NPs-Am nanoparticles were dissolved in 3.7 mL of Na_2CO_3/$NaHCO_3$ buffer (pH 8.5, 100 mM). This solution was mixed with 100 μL of a solution of FITC in DMSO (1 mg mL^{-1}, 1% mol), and the conjugation was carried out overnight at room temperature under stirring in the dark.

The functionalized nanoparticles were purified by dialysis using Spectrum™ Labs Spectra/Por™ dialysis membrane (MWCO 3500) for 48 hrs against NaCl 100 mM in Milli-Q water, and for other 48 hrs against Milli-Q water to remove any unreacted FITC. Finally, the product was freeze-dried for 48 hrs to obtain a dry yellow powder.

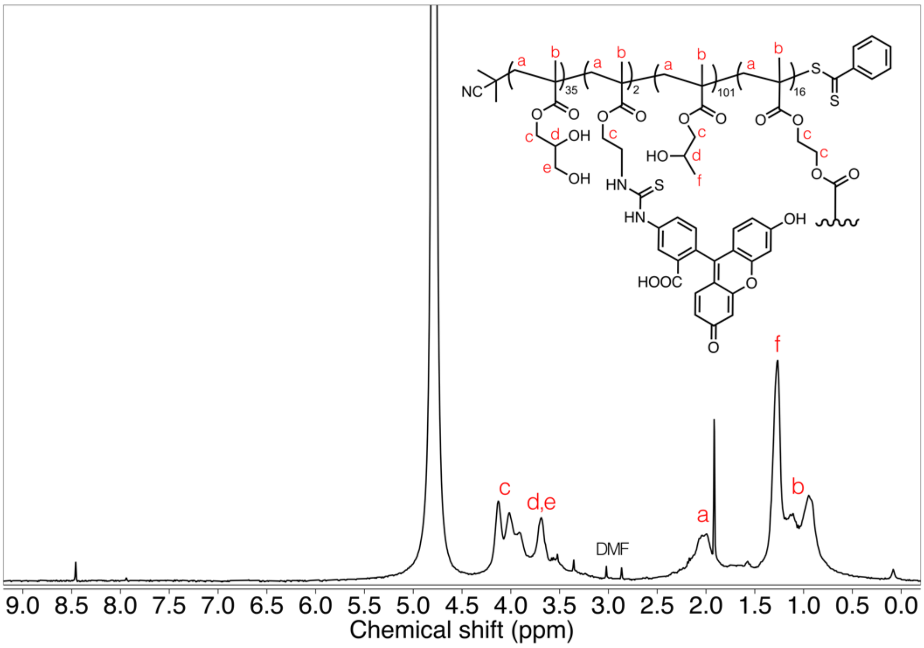

Figure S9. ^{1}H-NMR (400 MHz, D_2O) spectrum of FITC@NPs-Am in D_2O referenced against the peak at 4.79 ppm of HDO.

2.7 Poly(*N*-isopropylacrylamide-*co*-*N*-acryloxysuccinimide), p(NIPAM$_{51}$-AANHS$_3$)

Poly(*N*-isopropylacrylamide-*co*-*N*-acryloxy succinimide) was synthesised using a RAFT polymerization technique, adapting a previously established procedure.[4] NIPAM and AIBN were freshly recrystallized from hexane and methanol, respectively. NIPAM (927.1 mg, 8.2 mmol), AANHS (72.9 mg, 0.43 mmol), AIBN (5.1 mg, 30 μmol) and 4-cyano-4-(((propylthio)carbonothioyl)thio)pentanoic acid as RAFT agent (37.5 mg, 0.13 mmol) were dissolved in acetonitrile (4 mL) in a Schlenk tube equipped with a magnetic stirring bar, adding DMF (300 μL, 2.89 mmol) as internal standard to assess the monomer conversion *via* ^{1}H-NMR. The solution was purged from oxygen using a freeze-pump-thaw technique and the Schlenk tube was filled with argon and sealed. The polymerization was carried out at 65 °C for 5 hrs 30 min under stirring. The polymer was isolated by precipitation from hexane/diethyl ether (1:1 v/v%) as a crystalline light-yellow powder (758.0 mg, 76%). ^{1}H-NMR analysis of the purified polymer indicated a monomers conversion of 85%, a NIPAM DP of 51, an AANHS DP of 3, and an estimated M_n of 6,500 g mol^{-1}.

TD-GPC: *dn/dC* = 0.187 ± 0.011 mL g^{-1}; M_n = 9,865 ± 278 g mol^{-1}; M_w = 10,047 ± 286 g mol^{-1}; $Đ$ = 1.018 ± 0.003.

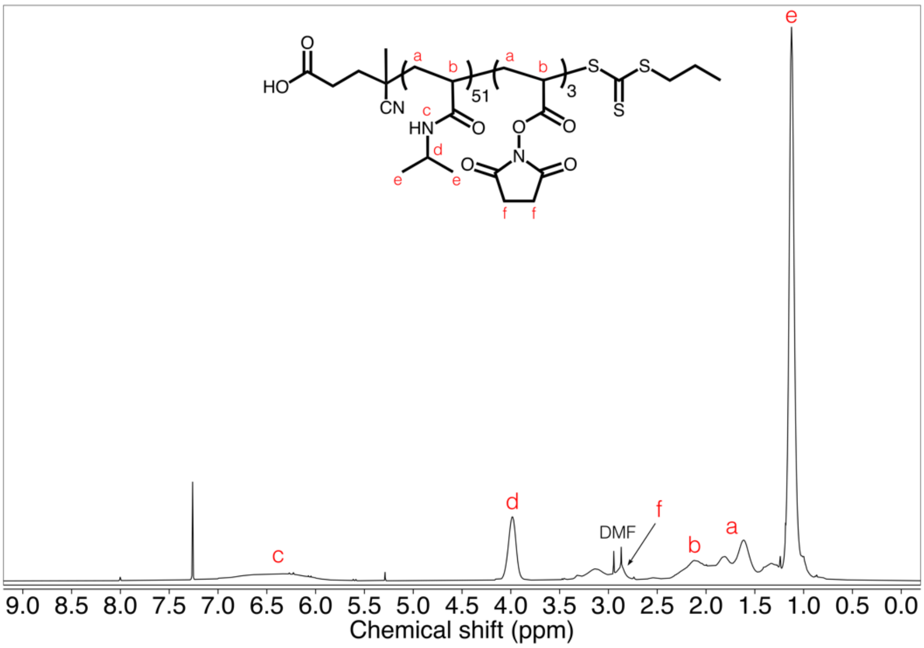

Figure S10. ^{1}H-NMR (400 MHz, $CDCl_3$) spectrum of p($NIPAM_{51}$-$AANHS_3$) polymer in $CDCl_3$ referenced against the peak of residual $CHCl_3$ at 7.26 ppm.

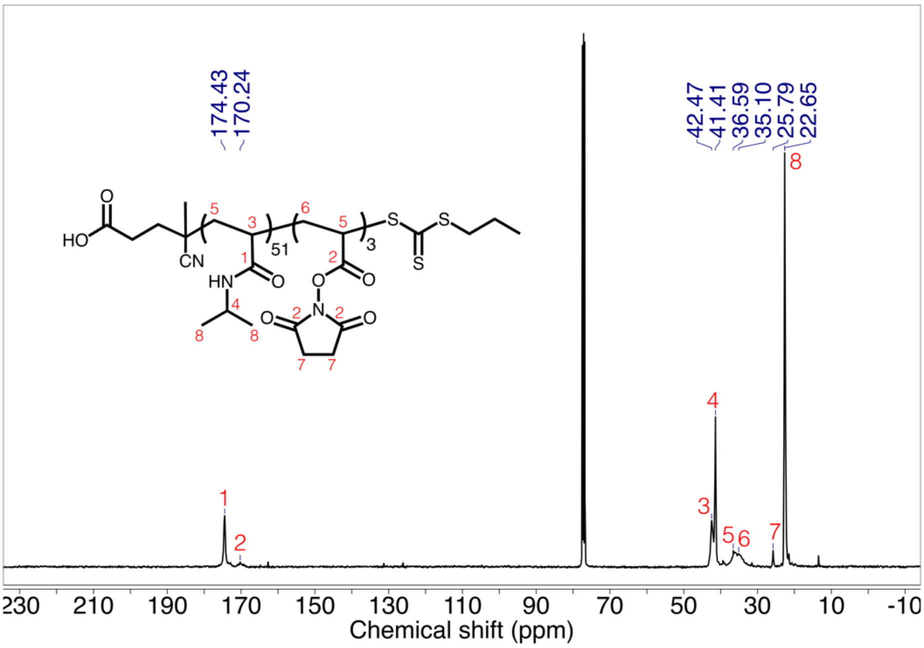

Figure S11. ^{13}C-NMR (100.5 MHz, $CDCl_3$) spectrum of p($NIPAM_{51}$-$AANHS_3$) polymer in $CDCl_3$ referenced against the peak of $CDCl_3$ at 77.16 ppm.

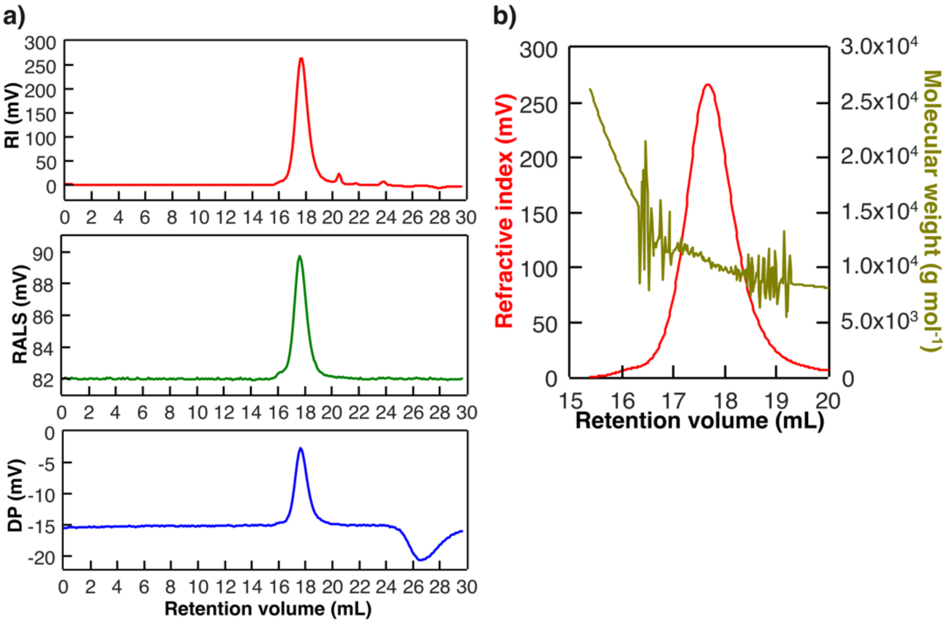

Figure S12. TD-GPC characterization of p(NIPAM$_{51}$-AANHS$_3$) polymer (3 mg mL^{-1} in NaNO$_3$ 0.1 M, pH 2.6). **(a)** Stacked response of the detectors as a function of the retention volume: refractive index (red plot), right angle light scattering (green plot), and viscometer (blue plot) traces. All three signals exhibit a single, well-defined peak eluting at the same retention volume, indicating a monomodal and narrow size distribution. The absence of high- or low-molecular weight tailing indicates negligible interaction between the polymer and the stationary phase of the columns. **(b)** Overlay of the refractive index detector response as a function of the retention volume (red plot) with the corresponding molecular weight calculated from the RALS detector (dark yellow). The molecular weight remains consistent across the elution peak, demonstrating the polymer's narrow dispersity (*Đ*) and confirming a controlled RAFT polymerization process.

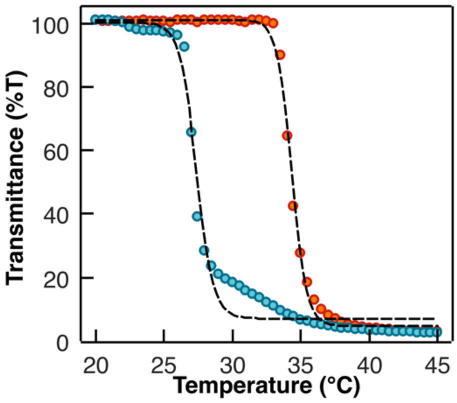

Figure S13. Characterization of thermoresponsive properties of p(NIPAM$_{51}$-AANHS$_3$) polymer (1 mg mL^{-1} in Milli-Q water). The temperature-dependent changes in transmittance (%T) were measured at 450 nm using a UV-Vis spectrophotometer in a temperature range from 20 to 45 °C. The transmittance change was monitored after heating (red points) and cooling (light blue points) and the LSCT estimated with Boltzmann sigmoidal fitting (dashed black curves). For the heating cycle, the fitting yielded an LCST (x_0) of 34 ± 1 °C and a slope factor (dx) of 0.55 ± 0.02 °C. For the cooling cycle, the fitting yielded an LCST (x_0) of 27 ± 1 °C and a slope factor (dx) of 0.73 ± 0.06 °C. For further details about Boltzmann fitting, see *Materials and Methods* section. The hysteresis that is observed in the cooling process corresponds to a complex coil-to-globule transition involving four distinct thermodynamically stable states.[5]

2.8 Functionalization of p(GMA_{35}-$AEMA_{2}$-$HPMA_{101}$-$EGDMA_{16}$) nanoparticles with p($NIPAM_{51}$-$AANHS_{3}$) polymer, p(NIPAM)@NPs-Am

In a vial, 10.3 mg of NPs-Am were dissolved in 5.0 mL of Na_2CO_3/$NaHCO_3$ buffer (pH 8.5, 100 mM). In a separate vial, 10.1 mg of p($NIPAM_{51}$-$AANHS_{3}$) polymer were dissolved in 5.0 mL of Na_2CO_3/$NaHCO_3$ buffer (pH 8.5, 100 mM). The two solutions were mixed, and the conjugation was carried out overnight at room temperature under stirring. The functionalized nanoparticles were purified by dialysis using Spectrum™ Labs Spectra/Por™ dialysis membrane (MWCO 14,000) for 48 hrs against Milli-Q water to remove any unreacted p($NIPAM_{51}$-$AANHS_{3}$) polymer. Finally, the product was freeze-dried for 48 hrs to obtain a white dry powder.

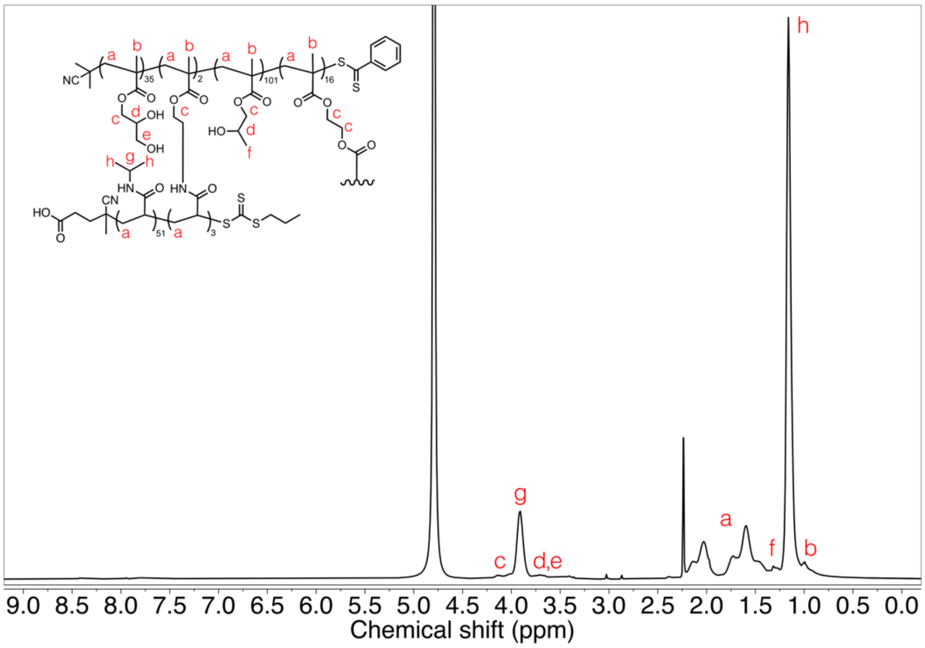

Figure S14. ^{1}H-NMR (400 MHz, D_2O) spectrum of (NIPAM)@NPs-Am in D_2O referenced against the peak at 4.79 ppm of HDO.

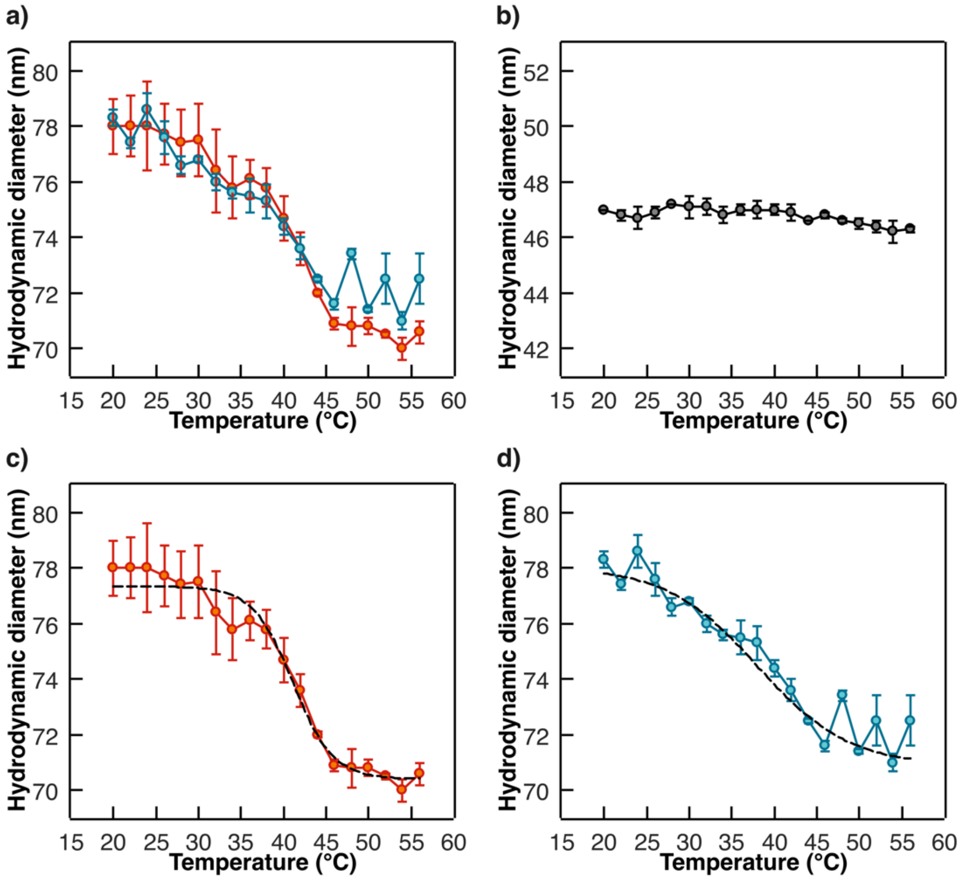

Figure S15. **(a)** Plots showing temperature-dependent DLS hydrodynamic diameter measurements (temperature range from 20 to 56 °C) of p(NIPAM)@NPs-Am; heating cycle (red plot), cooling cycle (light blue plot). **(b)** Same experiment performed on pristine NPs-Am (control experiment). All measurements were carried out using 0.2 mg mL^{-1} of nanoparticles in Milli-Q water. **(c)** Boltzmann sigmoidal fitting for LSCT estimation from the heating cycle in (a). By applying the fitting, an LCST (x_0) of 41 ± 1 °C and a slope factor (*dx*) of 3.84 ± 0.60 °C were calculated. **(d)** Boltzmann sigmoidal fitting of cooling cycle in (a) to test the reversibility of the temperature-dependent contraction. By applying the fitting, an LCST (x_0) of 37 ± 2 °C and a slope factor (*dx*) of 5.68 ± 2.03 °C were calculated. For further details about Boltzmann fitting, see *Materials and Methods* section. In contrast with the free p($NIPAM_{51}$-$AANHS_3$) in solution (Supplementary Figure S12), no hysteresis was observed, indicating a completely reversible coil-to-globule transition of the p(NIPAM)-based corona on the NPs-Am. All error bars: standard deviation on three consecutive measurements performed on the same sample.

2.9 Poly(*N*-isopropylacrylamide-*co*-*N*-acryloxysuccinimide), p($NIPAM_{17}$-$AANHS_1$)

Poly(*N*-isopropylacrylamide-*co*-*N*-acryloxysuccinimide) was synthesized *via* free radical polymerization. NIPAM and AIBN were used as purchased without recrystallization. In a vial equipped with a stirring bar, NIPAM (927.1 mg, 8.2 mmol), AANHS (72.9 mg, 0.4 mmol), and AIBN (70.8 mg, 0.4 mmol) were dissolved in acetonitrile (3 mL). DMF (300 µL, 2.89 mmol) was added as an internal standard to assess the monomer conversion *via* ^{1}H-NMR spectroscopy. The Schlenk tube was sealed, and the polymerization was carried out at 65 °C for 18 hrs under stirring. The polymer was isolated by precipitation from hexane/diethyl ether (1:1 v/v%) as a crystalline powder (965.0 mg, 90%). ^{1}H-NMR analysis of the purified polymer indicated a monomers conversion of 100%, a NIPAM DP of 17, an AANHS DP of 1, and an estimated M_n of 2,250 g mol^{-1}.

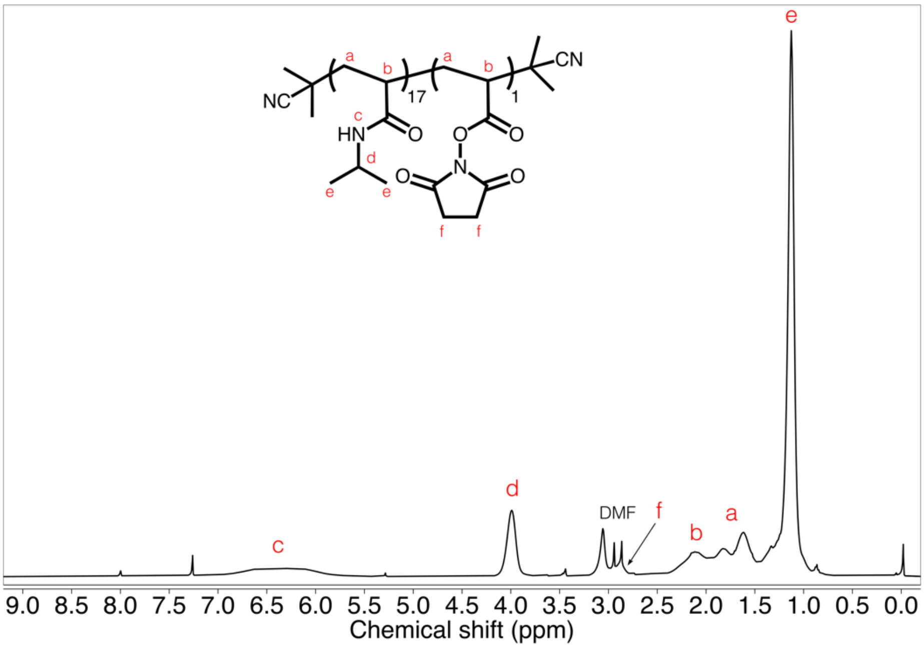

Figure S16. ^{1}H-NMR (400 MHz, $CDCl_3$) spectrum of p($NIPAM_{17}$-$AANHS_1$) polymer in $CDCl_3$ referenced against the peak of residual $CHCl_3$ at 7.26 ppm.

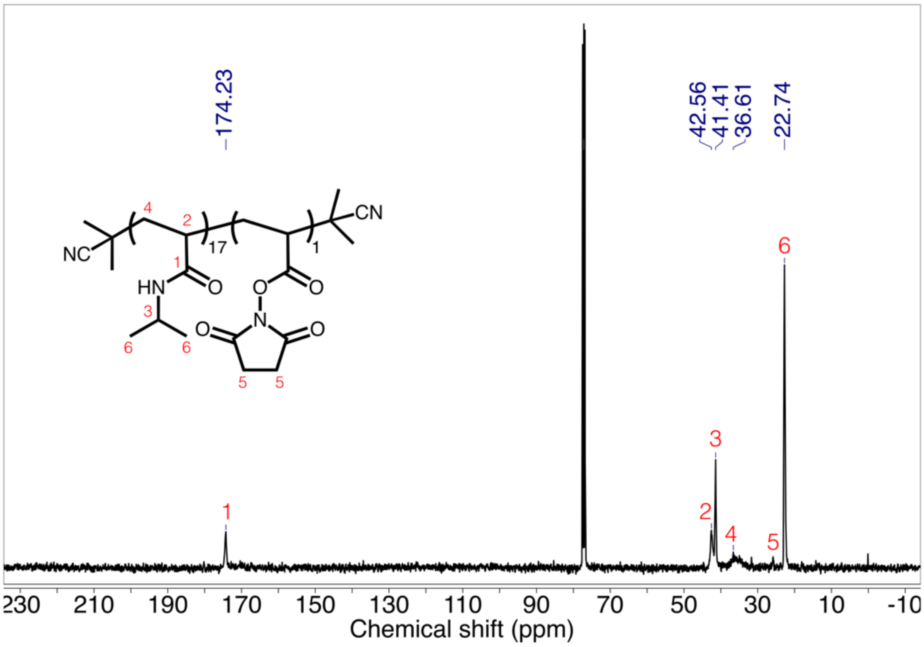

Figure S17. ^{13}C-NMR (100.5 MHz, $CDCl_3$) spectrum of p($NIPAM_{17}$-$AANHS_1$) polymer in $CDCl_3$ referenced against the peak of $CDCl_3$ at 77.16 ppm.

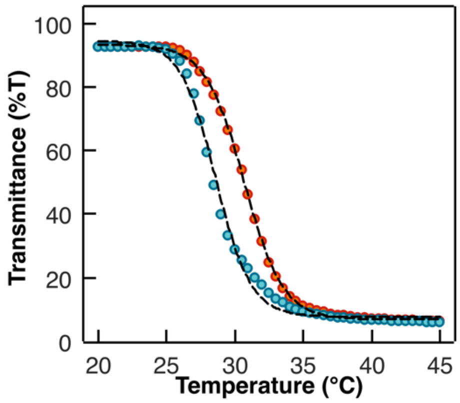

Figure S18. Characterization of thermoresponsive properties of p(NIPAM$_{17}$-AANHS$_1$) polymer (1 mg mL^{-1} in Milli-Q water). The temperature-dependent changes in transmittance (%T) were measured at 450 nm using a UV-Vis spectrophotometer in a temperature range from 20 to 45 °C. The transmittance change was monitored after heating (red plot) and cooling (light blue plot). The LSCT was estimated with a Boltzmann sigmoidal fitting (dashed black curves). For the heating cycle, the fitting yielded an LCST (x_0) of 31 ± 1 °C and a slope factor (*dx*) of 1.44 ± 0.02 °C. For the cooling cycle, the fitting yielded an LCST (x_0) of 29 ± 1 °C and a slope factor (*dx*) of 1.34 ± 0.05 °C. For further details about Boltzmann fitting, see *Materials and Methods* section. The hysteresis that is observed in the cooling process corresponds to a complex coil-to-globule transition involving four distinct thermodynamically stable states.[5]

3. Stability studies of NPs-Am in carbonate buffer for 48 hrs

In a vial, 20.2 mg of NPs-Am were dissolved in 20.2 mL of Na_2CO_3/$NaHCO_3$ buffer (pH 8.5, 100 mM) and were left for 48 hrs at room temperature under stirring. The NPs-Am were purified by dialysis against Milli-Q water to remove buffer salts using Spectrum™ Labs Spectra/Por™ dialysis membrane (MWCO 3,500) for 24 hrs. Finally, the product was freeze-dried for 48 hrs to obtain a white dry powder. DLS and ζ-potential before and after basic treatment measurements were performed in Milli-Q water.

Table S5. Characterization of NPs-Am before and after base-treatment with Na_2CO_3/$NaHCO_3$ buffer (pH 8.5, 100 mM).

Analysis	Pre-base treatment	Post-base treatment
Size-DLS (nm)	80.0 ± 1.1	65.9 ± 4.2
ζ-potential (mV)	+ 27.9 ± 1.9	- 23.4 ± 1.6
Kaiser Test (μmol g^{-1})	50 ± 10	4 ± 1

4. Preparation of Pickering emulsions and stable w/w colloidosomes

Pickering emulsions were prepared in a 1.8 mL glass vial by mixing 200 μL of a nanoparticles dispersion (5 mg mL^{-1}) in Na_2CO_3/$NaHCO_3$ buffer (pH 8.5, 100 mM) with an equal volume of 2-ethyl-1-hexanol oil solution (200 μL) gently added to the aqueous phase. The mixture was vigorously shaken for 15 sec to produce a white turbid dispersion. The resulting oil-in-water (o/w) Pickering emulsion was readily transferred into an Eppendorf tube to let it deposit prior to microscopy imaging. Greenfluorescent crosslinked colloidosomes were prepared using the same

experimental procedure described above with the exception that FITC (5 µL, 1 mg mL^{-1} in DMSO) and p(NIPAM$_{17}$-AANHS$_1$) crosslinker (0.1 mg, 20 µL in chloroform) were previously dissolved in the oil phase before layering it on the nanoparticles aqueous dispersion (200 µL). The resulting o/w Pickering emulsion was allowed to crosslink at 4 °C for 48 hrs. In order to transfer the crosslinked colloidosomes from the o/w biphasic system to water only, the colloidosomes were dialyzed at 4 °C with Spectrum™ Labs Spectra/Por™ dialysis membrane (MWCO 14000) against 70% ethanol (EtOH) in water for 5 hrs, then against 30% EtOH in water for 3 hrs, and finally against Milli-Q water overnight.

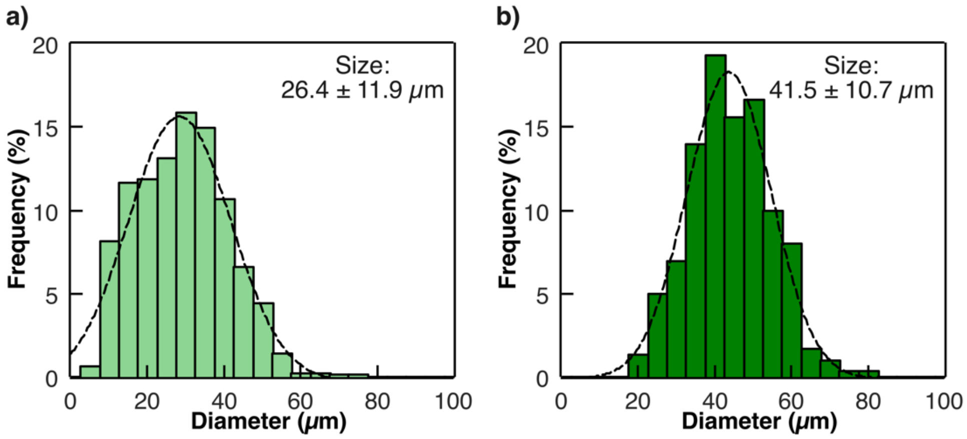

Figure S19. Plots showing size distributions of FITC-tagged and p(NIPAM$_{17}$-AANHS$_1$)-crosslinked NPs-Amo/w Pickering emulsions **(a)**, and of the corresponding colloidosomes transferred to water **(b)**. The dashed black curves represent the Gaussian fitting of data.

4.1 Permeability studies of crosslinked w/w colloidosomes

Experiments were performed by mixing 40 µL of colloidosome dispersion in Milli-Q water with 40 µL of FITC-labeled dextran solution (0.1 mg mL^{-1}, MW from 3 to150 kDa) in MilliQ water, following a previously established protocol.[4] Colloidosomes were incubated overnight in darkness with FITC-dextran at 4 °C. The permeability was described as the ratio of fluorescence intensity inside the colloidosome membrane (FI_{inside}) to outside the colloidosome membrane ($FI_{outside}$) against the molecular weight (MW) of FITC-dextran. All data are based on normalized fluorescence intensity values determined from confocal fluorescence microscopy images using ImageJ software.

4.2 Contractile temperature-dependent behaviour of crosslinked w/w colloidosomes

A colloidosomes dispersion in Milli-Q water was introduced into a hand-made microscope channel slide which was sealed with silicon grease and placed on the top of a heating plate installed on a fluorescence microscope mobile stage. The temperature was regulated through a thermostat, modified in a range from 25 to 45 °C (heating cycle, 2 °C steps) and from 45 to 25 °C (cooling cycle, 2 °C steps) and allowed to equilibrate for at least 15 min before acquiring the images with Axio Observer 7 (Zeiss) microscope. The sample temperature was measured by a thermocouple

thermometer directly positioned onto the channel slide. Changes in the colloidosomes volume were determined from fluorescence microscopy images using ImageJ software. Measurements were performed on at least ten different colloidosomes.

Bibliography

(1) Iannazzo, D.; Piperno, A.; Ferlazzo, A.; Pistone, A.; Milone, C.; Lanza, M.; Cimino, F.; Speciale, A.; Trombetta, D.; Saija, A.; Galvagno, S. Functionalization of Multi-Walled Carbon Nanotubes with Coumarin Derivatives and Their Biological Evaluation. *Org. Biomol. Chem.* **2012**, *10* (5), 1025–1031. https://doi.org/10.1039/c1ob06598j.

(2) Blanazs, A.; Ryan, A. J.; Armes, S. P. Predictive Phase Diagrams for RAFT Aqueous Dispersion Polymerization: Effect of Block Copolymer Composition, Molecular Weight, and Copolymer Concentration. *Macromolecules* **2012**, *45* (12), 5099–5107. https://doi.org/10.1021/ma301059r.

(3) Williams, M.; Penfold, N. J. W.; Armes, S. P. Cationic and Reactive Primary Amine-Stabilised Nanoparticles via RAFT Aqueous Dispersion Polymerisation. *Polym. Chem.* **2016**, *7* (2), 384–393. https://doi.org/10.1039/c5py01577d.

(4) Gobbo, P.; Patil, A. J.; Li, M.; Harniman, R.; Briscoe, W. H.; Mann, S. Programmed Assembly of Synthetic Protocells into Thermoresponsive Prototissues. *Nat. Mater.* **2018**, *17* (12), 1145–1153. https://doi.org/10.1038/s41563-018-0183-5.

(5) Lutz, J. F.; Akdemir, Ö.; Hoth, A. Point by Point Comparison of Two Thermosensitive Polymers Exhibiting a Similar LCST: Is the Age of Poly(NIPAM) Over? *J. Am. Chem. Soc.* **2006**, *128* (40), 13046–13047. https://doi.org/10.1021/ja065324n.
